# Design, Synthesis of New 1,2,4-Triazole/1,3,4-Thiadiazole with Spiroindoline, Imidazo[4,5-b]quinoxaline and Thieno[2,3-d]pyrimidine from Isatin Derivatives as Anticancer Agents

**DOI:** 10.3390/molecules27030835

**Published:** 2022-01-27

**Authors:** Ameen Ali Abu-Hashem, Sami A. Al-Hussain

**Affiliations:** 1 Heterocyclic Unit, Photochemistry Department, National Research Centre, Dokki, Giza 12622, Egypt; 2 Chemistry Department, Faculty of Science, Jazan University, Jazan 45142, Saudi Arabia; 3 Department of Chemistry, Faculty of Science, Imam Mohammad Ibn Saud Islamic University (IMSIU), Riyadh 13318, Saudi Arabia; sahussain@imamu.edu.sa

**Keywords:** thioxo-spiroindoline, isoindolinone, isatin, triazole, thiadiazole, hydrazine, thiazole, pyrimidine, imidazole, quinoxaline, anti-proliferative, cytotoxic activity

## Abstract

The current work aims to design and synthesis a new series of isatin derivatives and greatly enhances their cytotoxic activity. The derivatives 3-((bromophenyl) imino)-1-(morpholino (pyridine) methyl) indolin-2-one, 2-((oxoindoline) amino) benzoic acid, 3-(thiazolo-imino) indolinone, ethyl-2-((oxoindolin-3-ylidene)amino)-benzothiophene-3-carboxylate, 1-(oxoindoline)-benzo[4,5] thieno [2,3-d]pyrimidin-4(1H)-one, ethyl-2-(2-oxoindoline) hydrazine-1-carboxylate, N-(mercapto-oxo-pyrimidine)-2-(oxoindoline) hydrazine-1-carboxamide, N-(oxo-thiazolo[3,2-a] pyrimidine)-2-(oxoindolin-ylidene) hydrazine-carboxamide, 3-((amino-phenyl) amino)-3-hydroxy- indolinone, 3-((amino-phenyl) imino)-indolinone, 2-(2-((oxoindoline) amino) phenyl) isoindolinone, 2-(oxoindoline) hydrazine-carbothioamide, 5′-thioxospiro[indoline-3,3′-[1,2,4]triazolidin]-one, 5′-amino-spiro[indoline-3,2′-[1,3,4]thiadiazol]-2-one and 3-((2-thioxo-imidazo[4,5-b]quinoxaline) imino) indolinone were synthesized from the starting material 1-(morpholino (pyridine) methyl) indoline-2,3-dione and evaluated for their in vitro cytotoxic activity against carcinogenic cells. The new chemical structures were evidenced using spectroscopy (IR, NMR and MS) and elemental analysis. The results show that compounds imidazo[4,5-b]quinoxaline-indolinone, thiazolopyrimidine-oxoindoline, pyrimidine-oxoindoline-hydrazine-carboxamide, spiro[indoline-3,2′-[1,3,4] thiadiazol]-one and spiro[indoline-3,3′-[1,2,4]triazolidin]-one have excellent anti-proliferative activities against different human cancer cell lines such as gastric carcinoma cells (MGC-803), breast adenocarcinoma cells (MCF-7), nasopharyngeal carcinoma cells (CNE2) and oral carcinoma cells (KB).

## 1. Introduction

Recently, cancer has been spreading among people at a high rate. Thus, cancer is one of the main causes of death. In 2017, the death of nearly 9.6 million people due to different types of cancer made it the second leading cause of death, second only to cardiovascular diseases [1,2].

Hence, in the last decade, many researchers have worked to prepare various heterocyclic compounds that are used in the treatment of most cancer cells in the human body. For instance, isatin derivatives are an interesting category of heterocyclic molecules that have different biological and pharmacological activities [3].

In addition, spiro compounds have been showing distinguished pharmacological activity, especially in the chemistry of natural products. Many studies have been demonstrating the synthesis of spiro derivatives of isatin at the (C-3) position as α-methylene γ-butyrolactone spirocyclic, which is derived from isatin and is the proper core for optimization to identify new anticancer agents [4]. The spiro pyrazole-oxindole analogues are considered as valuable anticancer agents against human cancer cells [5].

Furthermore, the oxindolylidene acetate derivatives showed potent anticancer activities against a diversity of human cancer cell lines [6]. The indoline-2,3-dione moiety is a significant constituent of many pharmaceutically essential compounds such as sunitinib and hesperidin.

**Figure 1:**
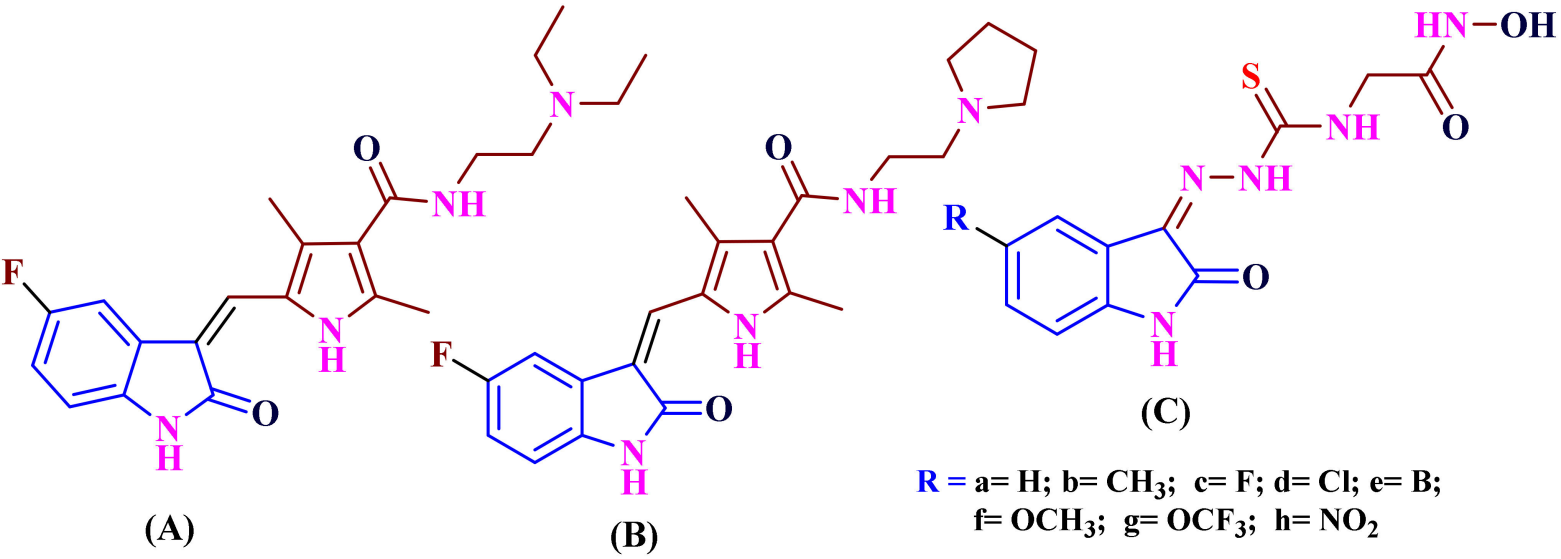
Anticancer drugs: Sunitinib (**A**), Toceranib phosphate (**B**), substituted isatin-hydroxamic acid (**C**) derived from isatin derivatives.

In a recent study, the researchers widely explored isatin derivatives in several clinically approved anticancer drugs, including *Sunitinib* (A), *Toceranib phosphate* (B), and *5-Substituted isatin hydroxamic acid* (C). These derivatives are used as histone deacetylase (HDAC enzyme) inhibitors and possess anti-proliferative activities against cervical tumor cell lines [7,8], shown in (Figure 1).

**Figure 2:**
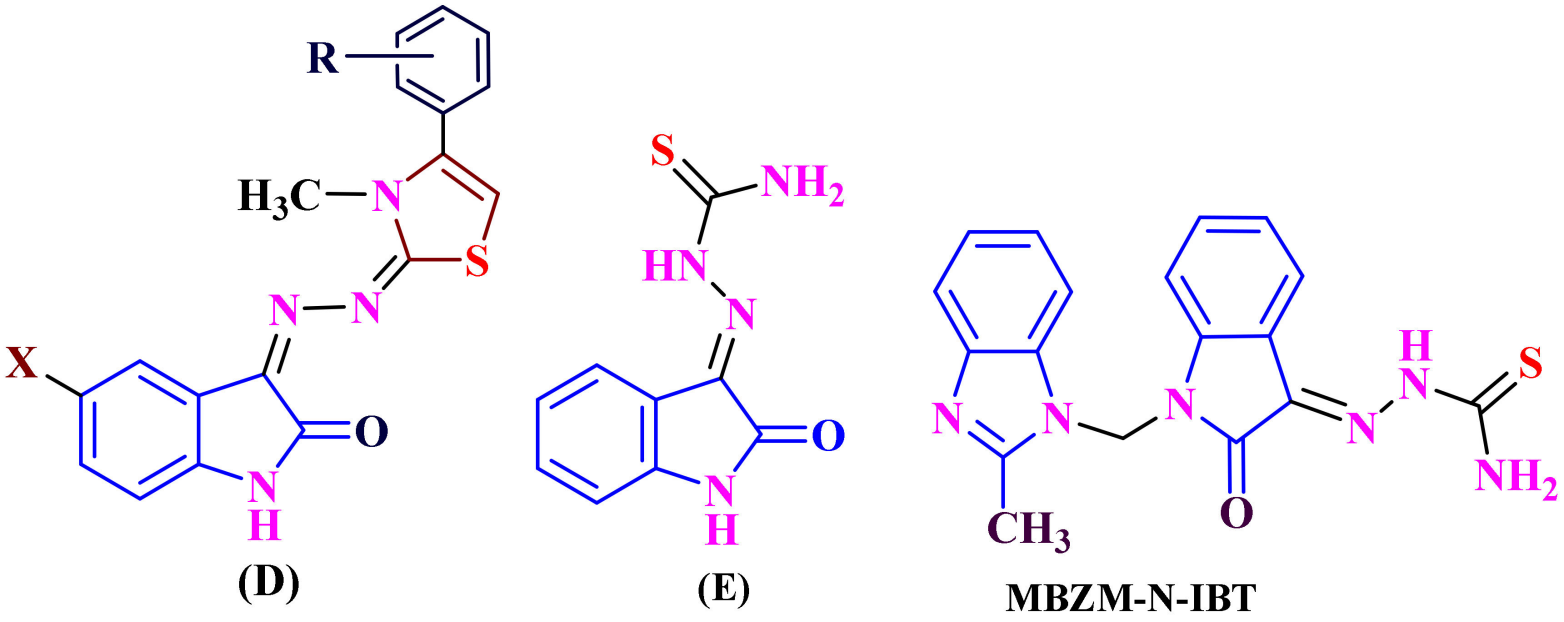
Anti-viral drugs: isatin-thiazoline hybrids (**D**), isatin-hydrazine-1-carbothioamide (**E**), isatin-β-thiosemicarbazone hybrids- imidazoles (MBZM- N-IBT) from isatin derivatives.

Moreover, scientific articles have confirmed that isatin derivatives such as the rhinoviruses [9], vaccinia virus [10], poxvirus [11], SARS virus [12,13] and Moloney leukemia virus [14] have antiviral activities. Recently, the *isatin-thiazoline hybrids* (D) have been shown to contain inhibitors of HIV-1 reverse transcriptase (RT) [15], *isatin-hydrazine- 1-carbothioamide* (E) has shown anti-CVB3 activity [16], and *isatin-β-thiosemicarbazone hybrids with imidazoles* (MBZM- N-IBT) have been show to possess the antiviral activities against the chikungunya virus (CHIKV) [17], shown in (Figure 2).

Furthermore, isatin’s importance has been promoted by the discovery of a number of new anti-tubercular agents [18], and a series of 1,2,3-triazole-isatin combined with ciprofloxacin represented anti-mycobacterial activity [19]. Furthermore, heterocyclic compounds containing heteroatoms such as *O*, *S* and *N* were considered effective inhibitors against the corrosion of steel in acidic media [20]. Hence, isatin derivatives and Mannich bases showed they are inhibitors against aluminum and steel corrosion [21].

Based on the above, isatin continues to be one of the more researched areas in synthetic and medicinal chemistry, aiding us in continuing our research work on the synthesis and design of biologically and pharmacological active heterocyclic compounds [22,23,24,25,26,27,28,29,30,31,32,33,34,35,36,37,38,39,40,41,42,43,44,45,46,47,48,49,50].

Thus, we introduce and evidence in this manuscript the synthesis of several isatin derivatives including morpholinopyridine-(thiazole-imino) indolinone; benzo[4,5]thieno [2,3-d]pyrimidinone; hydrazine-carboxylate; hydrazine-carboxamide; thiazolo[3,2-a]pyr- imidine; amino-iminoindolinone; thioxospiro[indoline-3,3′-[1,2,4]triazolidin]-one; spiro [indoline-3,2′-[1,3,4]thiadiazol]-2-one; and 3-((2-thioxo-imidazo[4,5-b]quinoxaline)imino) indolinone using recent procedures. The synthesized compounds were studied in vitro to evaluate their anticancer activity against different human cancer cell lines.

## 2. Results and Discussion

### 2.1. Synthesis

In order to perform one-pot synthesis using Mannich bases reaction [51,52,53], we synthesized 1-(morpholino (pyridin-4-yl) methyl) indoline-2, 3-dione **(2)** in excellent yield through stirring and refluxing a mixture of isatin **(1)** with freshly distilled secondary aliphatic amine as morpholine and isonicotinaldehyde in sodium ethoxide solution at room temperature under control (TLC). The IR spectrum compound **(2)** showed absorption bands at *ν* 1735 and 1680 cm^−1^ conforming to two carbonyl groups. ^1^H-NMR spectrums of **(2)** revealed multiplet signals at *δ* 2.60–3.05 ppm corresponding to eight protons of morpholine and one singlet at *δ* 6.72 ppm corresponding to one proton of methine proton.

The ^13^C-NMR spectrum of **(2)** displayed absorption signals at *δ* 51.2 and 58.8 ppm conforming to four carbon atoms of the (4CH_2_) morpholine and 95.4 ppm corresponding to one carbon atom of the methine proton (CH) group.

In continuation of our research plan in exploring the reactivity of isatin derivatives, we were interested in preparing 3-substituted-indolin-2-one, as expected as anticancer scaffolds. Therefore, syntheses of **(3–5)** started from 1- (morpholino (pyridin-4-yl) methyl) indoline-2,3-dione **(2)** through activation of the C_3_-substituted isatin, which is a carbonyl group, as a good leaving group with the exit water molecule as a requirement for the subsequent SNAr (nucleophilic aromatic substitution) reaction to occur with weak *N*-nucleophiles such as NH_2_-heterocycles. Moreover, compound **(2)** reacted with 4-bromo aniline in methanolic potassium hydroxide solution to yield 3-((4-bromophenyl) imino)-1- (morpholino (3yridine-4-yl) methyl) indolin-2-one **(3)**.

Furthermore, condensation of compound **(2)** with 2-aminobenzoic acid and anhydrous potassium carbonate in dry dimethylformamide afforded 2-((1-(morpholino (pyridin3-4-yl) methyl)-2-oxoindolin-3-ylidene) amino) benzoic acid **(4)**.

In addition, condensation of compound **(2)** with 2-aminothiazole and anhydrous potassium carbonate in dry dimethylformamide yielded 1-(morpholino (3yridine-4-yl) methyl)-3-(thiazol-2-ylimino) indolin-2-one **(5)**. The IR spectrum of **(3)** showed absorption bands at *ν* 1688 cm^−1^ for the one carbonyl group, and the ^13^C-NMR spectrum of **3** revealed absorption signals at *δ* 51.5 and 58.9 ppm corresponding to four carbon atoms of the (4CH_2_) morpholine, at *δ* 94.7 ppm corresponding to one carbon atom of the methine proton (CH) group and at *δ* 164.1 ppm conforming to one carbonyl group.

Likewise, the IR spectrum of **(4)** indicated absorption bands at *ν* 3750 cm^−1^ for the one hydroxyl group and at *ν* 1740 and 1688 cm^−1^ for two carbonyl groups, and the ^1^H-NMR spectrum of **(4)** evidenced one singlet signal at *δ* 12.60 ppm for the one proton of (OH) group (D_2_O exchangeable). The ^13^C-NMR spectrum of **(4)** exposed absorption signals at *δ* 52.2 and 59.1 ppm matching four carbon atoms of the (4CH_2_) morpholine, at *δ* 95.3 ppm corresponding for one carbon atom of the methine proton (CH) group and at *δ* 164.7 and 167.1 ppm corresponding to two carbonyl groups.

Similarly, ^1^H-NMR spectrums of **(5)** discovered multiplet signals at *δ* 2.72–3.18 ppm conforming to eight protons of morpholine, one singlet at *δ* 6.67 ppm corresponding to one proton of methine proton and two doublet signals at *δ* 7.78 and 7.85 ppm corresponding for two protons of (*J* = 6.92 Hz) thiazole ring. The ^13^C-NMR spectrum of **(5)** demonstrated absorption signals at *δ* 52.5 and 59.7 ppm corresponding to four carbon atoms of the (4CH_2_) morpholine, at 94.4 ppm corresponding to one carbon atom of the methine proton (CH) group and at *δ* 168.2 ppm conforming to one carbonyl group.

**Scheme 1:**
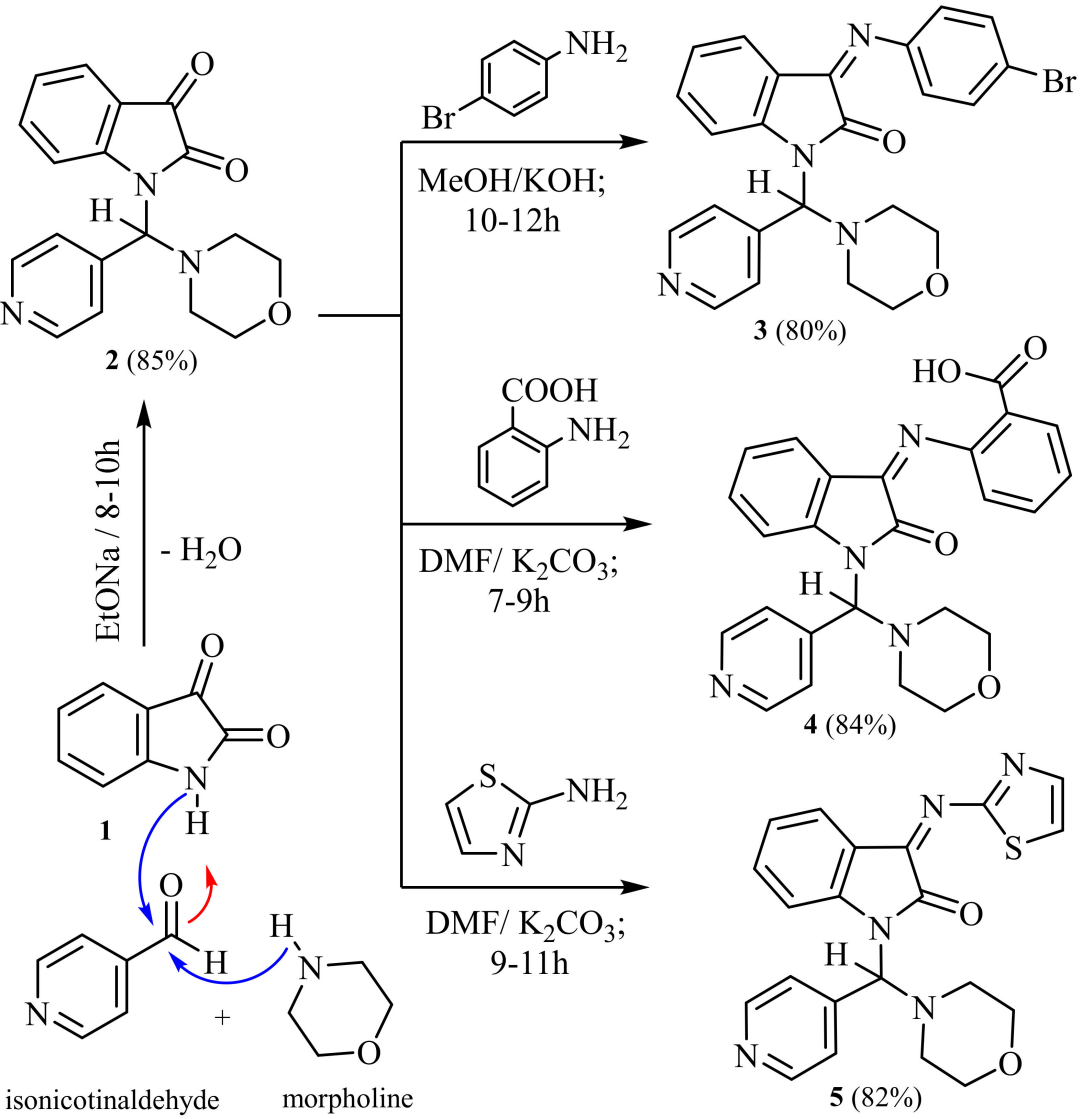
Synthesis of 1-(morpholino (pyridine) methyl)-2-oxoindoline) amino) and thiazole derivatives *via* condensation and Mannich bases reaction.

The mass spectra of **(2**, **3**, **4** and **5)** evidenced molecular ion peaks at *m*/*z* = 323 (M^+^, 100%), 477 (M^+^, 100%), 442 (M^+^, 100%) and 405 (M^+^, 100%), respectively. IR, NMR, MS and elemental analysis are elucidated in the experimental section for new compounds and shown in (Scheme 1).

Furthermore, we synthesized isatin with benzo[4,5]thieno[2,3-d]pyrimidine, hydrazine-1-carboxamide and thiazolo[3,2-a]pyrimidine moieties by nucleophilic aromatic substitution and condensation reactions and study of the cytotoxicity of synthesized compounds against human different carcinoma cell lines in vitro.

Furthermore, the compound **(2)** reacted with ethyl 2-amino-4,5,6,7-tetrahydro benzo[b]thiophene-3-carboxylate in refluxing dimethylformamide with the presence of potassium carbonate afforded ethyl-2-((1-(morpholino (pyridin-4-yl) methyl)-2-oxoind- olin-3-ylidene) amino)-4,5,6,7-tetrahydrobenzo[b] thiophene-3-carboxylate **(6)**. The IR spectrum of **(6)** indicated the presence of absorption bands at *ν* 1775 and 1682 cm^−1^ conforming for two carbonyl groups. The ^1^H NMR spectrum of **(6)** displayed multiplet signals at *δ* = 1.08–1.15 ppm for eight protons of (4CH_2_, cyclohexane), multiplet signals at *δ* = 2.75–3.22 ppm for eight protons of (4CH_2_, morpholine), and one singlet signal at *δ* = 6.62 ppm for one proton of methine proton (CH) group.

Furthermore, the latter compound **(6)** reacted with formamide in dimethylformamide with the presence of potassium carbonate for a long time under control (TLC) to yield 1-(1-(morpholino(pyridin-4-yl)methyl)-2-oxoindolin-3-yl)-5,6,7,8-tetrahydrobenzo [4,5]thieno[2,3-d]pyrimidin-4(1H)-one **(7)**. The 1H-NMR spectrum of **(7)** exhibited two singlets at δ = 5.10 and 8.15 ppm, corresponding to the one proton of pyrrole and one proton of pyrimidine ring, respectively.

Moreover, condensation of compound **(2)** with ethyl hydrazinecarboxylate in boiling dimethylformamide and potassium carbonate produced Ethyl-2-(1-(morpholino (pyridin-4-yl) methyl)-2-oxoindolin-3-ylidene) hydrazine-1-carboxylate **(8)**. The IR spectrum of **8** showed the presence of broad band absorption at *ν* 3325 cm^−1^ for one (NH) group and two peaks in absorption at *ν* 1691 and 1684 cm^−1^ for two carbonyl groups, and the ^1^H-NMR spectrum of **(8)** displayed a broad singlet at *δ* 10.50 ppm corresponding to the one proton of (NH) group exchangeable with D_2_O.

Furthermore, the treatment of the same compound **(8)** with 6-amino-2-mercapto- pyrimidin- 4(3*H*)-one in refluxing and stirring dimethylformamide and an amount of TEA (0.5 mL) as catalysis produced *N*-(2-mercapto-6-oxo-1,6-dihydropyrimidin-4-yl)-2- (1-(morpholi- no (pyridin-4-yl) methyl)-2-oxoindolin-3-ylidene) hydrazine-1-carboxami- de **(9)**. The IR spectrum of **(9)** displayed three peaks in broad band absorption at *ν* 3330–3270 cm^−1^ corresponding to three (3NH) groups and three peaks in absorption at *ν* 1695, 1688 and 1682 corresponding to three carbonyl groups, and ^1^H-NMR spectrum of **(9)** exhibited three broad singlets at *δ* 9.90, 10.80 and 11.70 ppm corresponding to the three protons of three (3NH) exchangeable with D_2_O.

Otherwise, condensation of the later compound **(9)** with chloroacetic acid in a mixture of glacial acetic acid and glacial acetic anhydride in the presence of anhydrous sodium acetate under stirring and refluxing led to *N*-(3,5-dioxo-2,3-dihydro- 5*H*-thiazolo[3, 2-a]pyrimidin-7-yl)-2-(1-(morpholino(pyridin-4-yl)methyl)-2-oxoindolin-3-ylidene)hydr- azine-1-carboxamide **(10)**. IR spectrum of **(10)** showed absorption broadband at *ν* 3325–3280 cm^−1^ conforming to two (2NH) groups and four peaks at *ν* 1691, 1686, 1681 and 1675 cm^−1^ for four carbonyl groups, and ^1^H-NMR spectrum of **(10)** exposed two broad singlets at *δ* 10.10 and 11.60 ppm corresponding to the two protons of two (2NH) groups exchangeable with D_2_O.

**Scheme 2:**
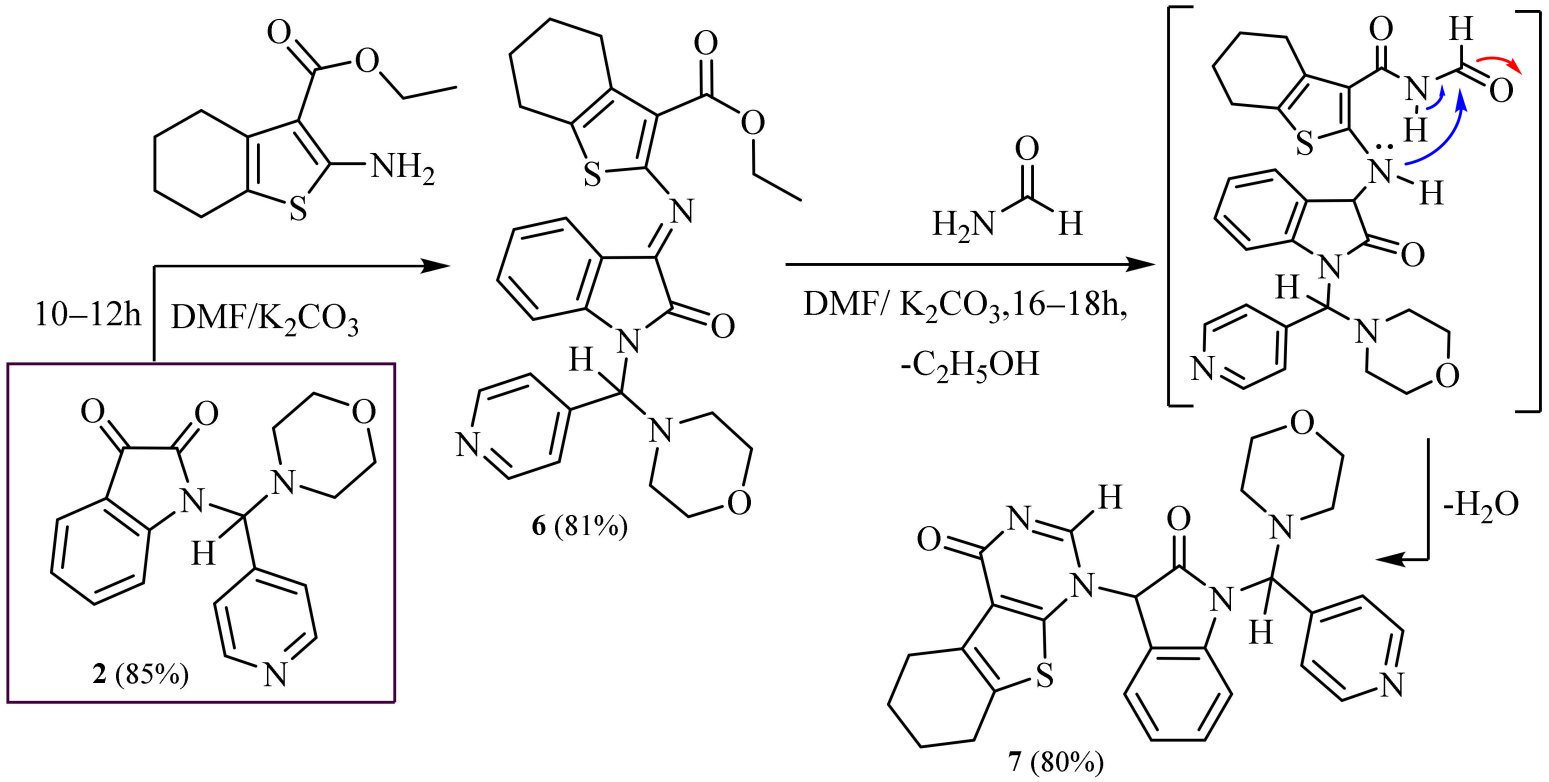
Synthesis of 2-oxoindoline- benzo [4,5] thieno [2,3-d] pyrimidinones.

**Scheme 3:**
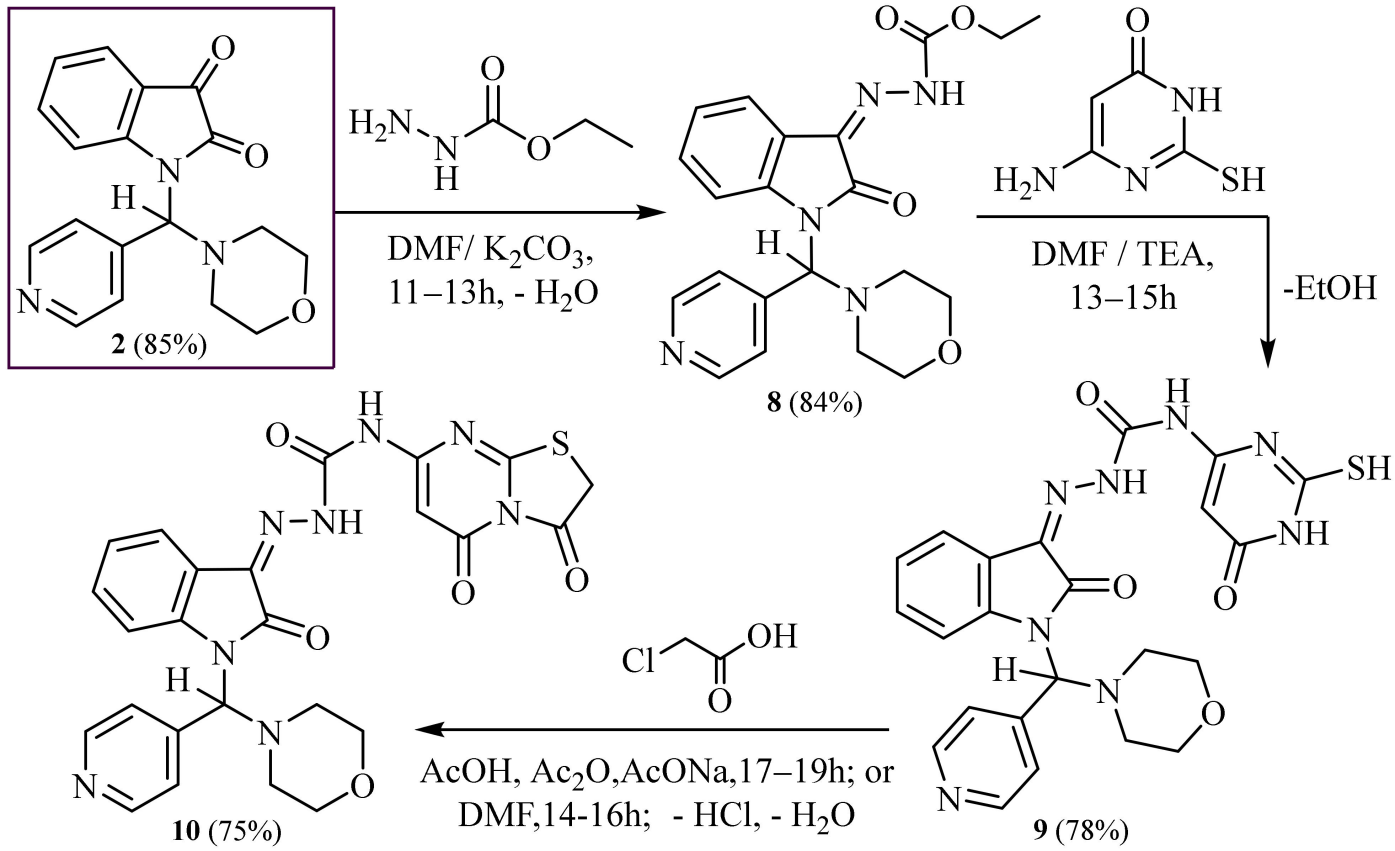
Synthesis of thiazolo [3,2-a] pyrimidine,-hydrazine-1-carboxamide with isatin.

MS spectrum of **(6**, **7**, **8, 9** and **10)** revealed molecular ion peaks at *m*/*z* = 530 (M^+^, 100%), 513 (M^+^, 95%), 409 (M^+^, 100%), 409 (M^+^, 100%) and 546 (M^+^, 92%), respectively. Correct new chemical structures were approved of through spectroscopic, IR, ^1^H, ^13^C, -NMR, mass spectra and elemental analysis, shown in (Exp. Part and Scheme 2 and Scheme 3).

We report here the synthesis of new 3-((2-aminophenyl) amino)-indolin-2-one, 3-((2-amino-phenyl) imino)-indolin-2-one and 2-((amino) phenyl) isoindoline-1, 3-dione and describe their anticancer properties.

Furthermore, the reaction of **(2)** with benzene-1, 2-diamine in methanol and diethyl amine with stirring and refluxing afforded 3-((2-aminophenyl) amino)-3-hydroxy-1-(morpholino (pyridin-4-yl) methyl) indolin-2- one **(11)**. Its IR of **(11)** shows three broad peaks at *ν* 3415, 3370 and 3330 cm^−1^ corresponding to (NH_2_), (OH) and (NH) groups, respectively, and a sharp intensity in one peak at *ν* 1682 cm^−1^ corresponding to one carbonyl group. The ^1^H-NMR spectrum of **(11)** contains three broad signals at *δ* 6.25, 11.10 and 12.60 ppm for two protons (NH_2_), one proton (NH) and one proton (OH) groups exchangeable with D_2_O, respectively.

Furthermore, stirring and refluxing of the same compound **(11)** in glacial acetic acid with concentrated HCl under (TLC) control afforded 3-((2-aminophenyl)imino)-1- (morpholi- no (pyridin-4-yl) methyl) indolin-2-one **(12)** in good yield. The IR spectrum compound (**12)** exhibited absorption bands at *ν* 3410 cm^−1^ corresponding to NH_2_ group and1684 cm^−1^ corresponding to one carbonyl group. ^1^H-NMR spectrum of **(12)** revealed one singlet at *δ* 6.35 ppm conforming to two protons of NH_2_ group (D_2_O exchangeable).

Moreover, treatment of the latter compound **(12)** with phthalic anhydrides in absolute methanol containing a few drops of glacial acetic acid afforded 2-(2-((1-(morpho- lino (pyridin-4-yl) methyl)-2-oxoindolin-3-ylidene) amino) phenyl) isoindoline-1, 3-dione **(13)** in good yield. IR spectrum of **(13)** showed absorption bands at *ν* 1688, 1682 and 1677 cm^−1^ for three carbonyl groups. The ^13^C-NMR spectrum of (**13)** exhibited absorption signals at *δ* 52.3 and 59.1 ppm, corresponding to four carbon atoms of (4CH_2_, morpholine), at *δ* 93.8 ppm for methine proton (CH) group and at *δ* 166.1, 168.5 ppm for three carbon atoms of the three carbonyl groups.

**Scheme 4:**
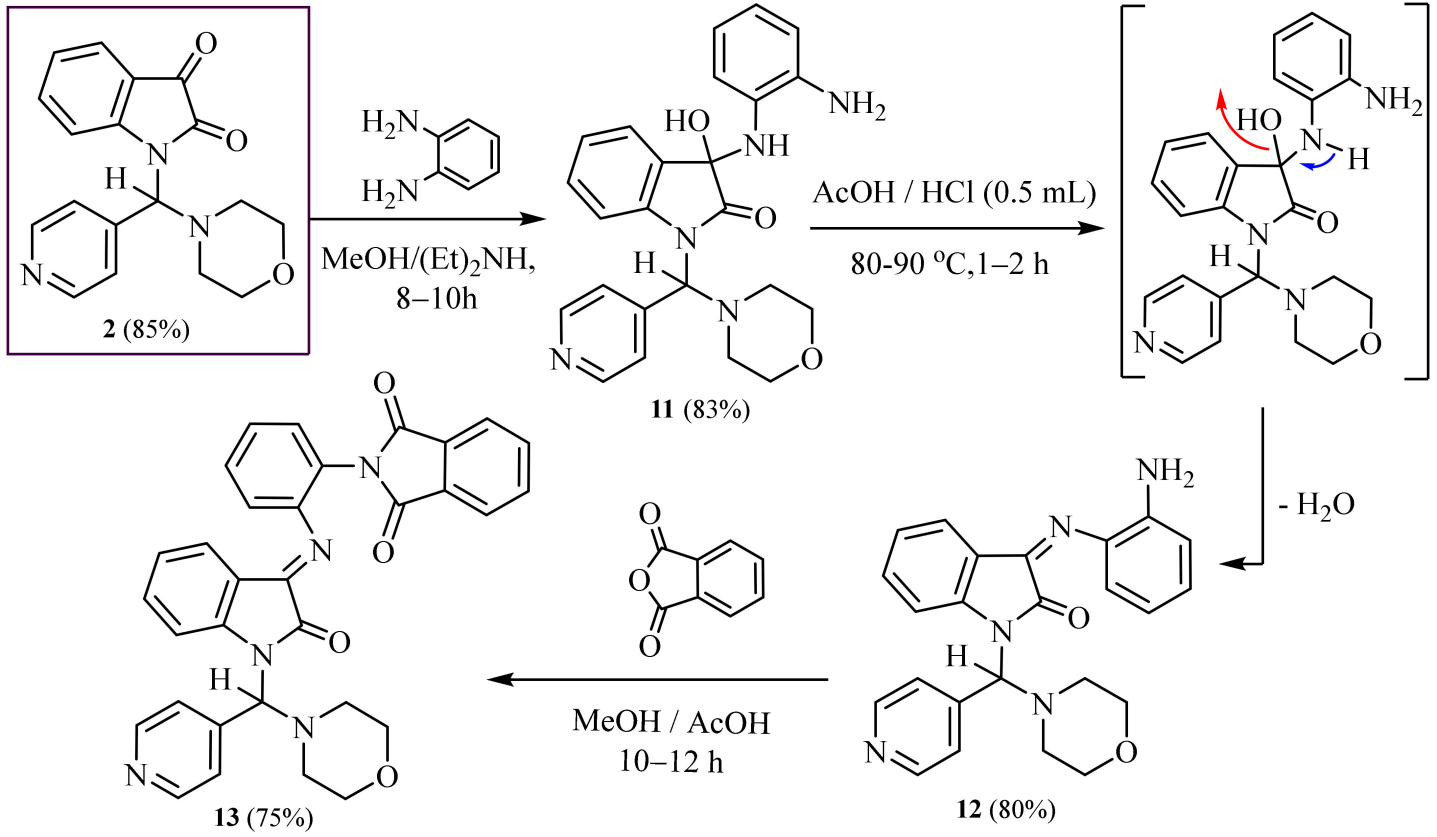
Synthesis of amino, imino,-phenyl and isoindoline-dione linked with indolinones.

The mass spectra of **(11**, **12** and **13)** evidenced molecular ion peaks at *m*/*z* = 431 (M^+^, 90%), 413 (M^+^, 100%) and 543 (M^+^, 100%), respectively. IR, NMR, MS and elemental analysis were explicated in the experimental section for all compounds and shown in (Scheme 4).

Herein we describe the synthesis and design of a series of isatin derivatives, hydrazine-1- carbothioamide; thioxospiro [indoline-3,3′-[1,2,4] triazolidin]-one; spiro [indoline-3,2′-[1,3,4] thiadiazol]-one and 3-((2-thioxo-imidazo [4,5-b] quinoxaline) imino) through reaction of thiosemicarbazide with isatin moieties.

Furthermore, the thiosemicarbazide with 1-(morpholino (pyridine) methyl) isatins **(2)** in glacial acetic acid was heated and refluxed to produce the target of 2- (1- (morpholino (pyridin-4-yl) methyl)-2-oxoindolin-3-ylidene) hydrazine-1- carbothioamide **(14)** as key starting material to synthesize many heterocyclic compounds. The IR spectrum of compound **(14)** exhibited absorption bands at *ν* 3430 and 3320 cm^−1^ corresponding to NH_2_ and NH groups, *ν* 1678 cm^−1^ for one carbonyl group and 1475 cm^−1^ for one (C=S) group. ^1^H-NMR spectrum of **(14)** demonstrated one singlet at *δ* 6.61 ppm corresponding to one proton of methine proton and two singlets at *δ* 6.90 and 11.35 ppm corresponding to the two protons of (NH_2_) and one proton of (NH) groups (D_2_O exchangeable), respectively.

Additionally, a new one-pot multi-component reaction for the perfect selective synthesis of the various spiro-indolines-based frameworks has been described. Thus, the multicomponent reaction involves the compound isatin **(2)** and thiosemicarbazide in glacial acetic acid under reflux for a long time to form 1-(morpholino (pyridin-4-yl) methyl)-5′-thioxospiro [indoline-3,3′-[1,2,4] triazolidin]-2-one **(15)**.

Furthermore, the same compound **(15)** can be prepared in another way, which is to use cycloaddition reactions of the latter compound **(14)** in dry chloroform or glacial acetic acid with a few drops of triethylamine as catalysis for a long time under reflux and (TLC) control. The IR spectrum compound **15** showed absorption bands at *ν* 3350–3230 cm^−1^ compatible with three (3NH) groups and *ν* 1688 cm^−1^ conforming to one carbonyl group and *ν* 1450 cm^−1^ for one (C=S) group.

^1^H-NMR spectrum of **15** revealed three broad singlets at *δ* 10.10, 10.40 and 11.55 ppm corresponding to three protons of (3NH) groups (D_2_O exchangeable). The ^13^C-NMR spectrum of **15** showed absorption signals at *δ* 97.1 ppm conforming to one spiro-carbon atom of (1C, 5′-thioxospiro[indoline-3,3′-[1,2,4] triazolidin]-2-one) and *δ* 168.2 ppm for one carbon atom of the one carbonyl group.

In addition, 5′-amino-1-(morpholino (pyridin-4-yl) methyl)-3′*H*-spiro[indoline-3,2′-[1,3,4]thiadiazol]-2-one **(16)** was prepared separately using compound **(14)** and iodine with potassium carbonate in boiling dioxane or DMF for long periods of time.

Another method for synthesizing a compound **(16)** is refluxing of compound (**14)** in ethanol containing a few drops of triethylamine as catalysis under (TLC) control. The IR spectrum of **(16)** displayed absorption bands at *ν* 3420 and 3335 cm^−1^ matching NH_2_ and NH groups and *ν* 1682 cm^−1^ for one carbonyl group. ^1^H-NMR spectrum of **(16)** proved one singlet at *δ* 6.85 ppm corresponding to two protons of (NH_2_) and 10.25 ppm corresponding to one proton of (NH) groups exchangeable with D_2_O. The ^13^C-NMR spectrum of (**16)** indicated absorption signals at *δ* 78.5 ppm corresponding to one spiro-carbon atom of (1C, 3′*H*-spiro[indoline-3,2′-[1,3,4]thiadiazol]-2-one) and *δ* 167.5 ppm for one carbon atom of the one carbonyl group.

Moreover, 1-(morpholino (pyridin-4-yl) methyl)-3-((2-thioxo-2,3-dihydro-1*H*-imid- azo [4,5-b] quinoxalin-1-yl) imino) indolin-2-one **(17)** was synthesized by nucleophilic aromatic substitution reactions of compound (**14)** with 2,3-dichloro-quinoxaline by refluxing in absolute ethanol in the presence of triethylamine as catalysis. The IR spectrum of (**17)** exposed absorption bands at *ν* 3340 cm^−1^ corresponding to NH group, 1686 cm^−1^ compatible with one carbonyl group and *ν* 1480 cm^−1^ conforming to one (C=S) group. ^1^H-NMR spectrum of (**17)** revealed one singlet at *δ* 10.60 ppm conforming to one proton of (NH) group (D_2_O exchangeable).

**Scheme 5:**
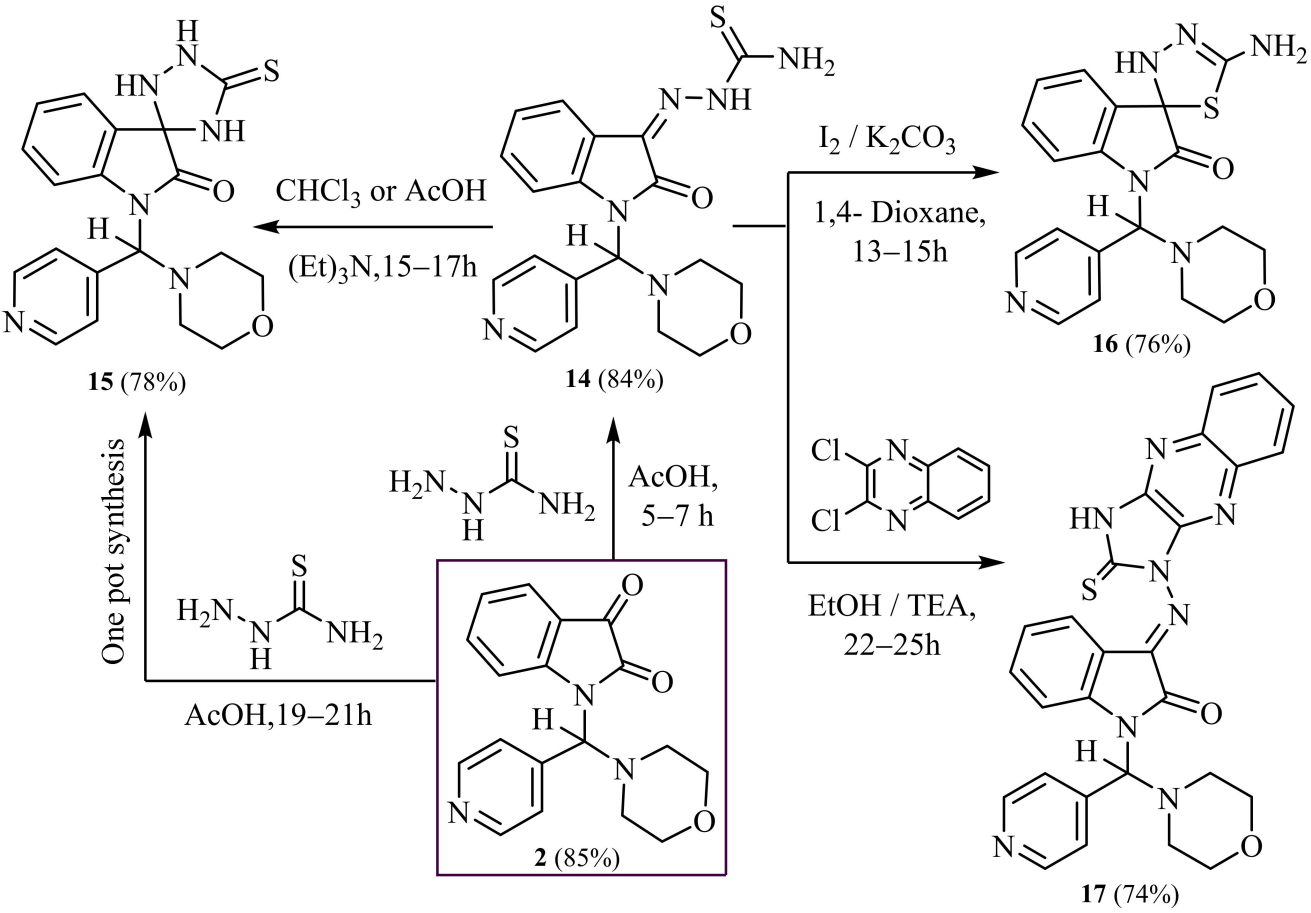
Synthesis of 1,2,4-triazole, 1,3,4-thiadiazole, -spiroindolinone and imidazo [4,5-b] quinoxaline-indolinones.

MS spectrum of **(14**, **15**, **16** and **17)** displayed molecular ion peaks at *m*/*z* = 396 (M^+^, 100%), 396 (M^+^, 94%), 396 (M^+^, 90%) and 522 (M^+^, 97%), respectively. Correct new chemical structures were evidenced via spectroscopic, infrared, nuclear magnetic resonance, mass spectra and elemental analysis, shown in (Exp. Part and Scheme 5).

### 2.2. Pharmacological Activities

#### 2.2.1. Anticancer Screening

##### In Vitro Cytotoxic Activity

The cytotoxic activities of new isatin derivatives were measured using the MTT colorimetric method by Mosmann (1983) [22,23,24,25,26,27,28,29,30,31,32,33,54] against four cancer cell lines: **MGC-803** cells are drug-sensitive human gastric carcinoma cells; **MCF-7** cells are drug-sensitive human breast adenocarcinoma cells; **CNE2** cells are drug-sensitive human nasopharyngeal carcinoma cells; and **KB** cells are drug-sensitive human oral carcinoma cells.

The IC_50_ values are summarized in (Table 1) and compared to 5-Fluorouracil as a positive control. IC_50_ values are located over a wide range of concentrations, from 9 μM to over 50 μM, showing a significant disparity in the cytotoxicity of the isatin compounds of various cancer cell lines.

The cytotoxic effects are cell-line-dependent, in the study of the final results shown in (Table 1) through the effect of isatin derivatives against the types of human carcinoma cell lines. Some of the compounds such as **17**, **10**, **9**, **16** and **15** gave the highest anticancer activity; the same compounds **17**, **10**, **9**, **16** and **15** represented the best effect of cytotoxicity and exceeded the drugs of 5-Fluorouracil positive control.

Moreover, the compounds **17**, **10**, **9**, **16** and **15** have the best activities against all carcinoma cell lines in the following: the IC_50_ compounds **17**, **10**, **9**, **16** and **15**; the **MGC-803** (9.7, 9.8, 9.9, 10.1, 10.3 μM); the **MCF-7** (9.6, 9.7, 9.8, 10.0, 10.2 μM); the **CNE2** (9.5, 9.6, 9.7, 9.9, 10.1 μM); and the **KB** (9.4, 9.5, 9.6, 9.8, 9.9 μM). Furthermore, some of the compounds that moderate resulted in activities against cancer cells such as **7**, **13**, **6** and **5**. Further, the rest of the compounds in this manuscript have a weak effect against cancer cells. All results concerning the compounds’ anticancer activities in vitro are shown in (Table 1).

#### 2.2.2. Structural Activity Relationship (SAR)

Isatin derivatives presented good antitumor activities against human cancer cell lines such as gastric, breast, nasopharyngeal, oral, lung and leukemia [22,23,24,25,26,27,28,29,30,31,32,33,55]. Furthermore, through the results we obtained, we can discuss the relationship of the chemical structures (SARs) of the synthesized compounds and their effect on the different types of cancer cell lines via the cytotoxicity activity of these compounds as follows:

**(I)** these compounds **17**, **10**, **9**, **16** and **15** (Table 2), exhibited excellent anticancer activity and some of the compounds (**7**, **13**, **6** and **5**) showed good anticancer activity in vitro. This agrees with previous scientific studies, because it includes many heterocyclic compounds possessing pharmacological activities such as imidazo[4,5-b]quinoxaline- iminoindolinone, *N*-thiazolo[3,2-a]pyrimidine-oxoindoline, *N*-(pyrimidine)-oxoindoline- hydrazine-1-carboxamide, spiro[indoline-3, 2′-[1,3,4] thiadiazol]-2-one, thioxospiro[ind- oline-3, 3′-[1,2,4] triazolidin]-one, oxoindoline-benzo[4,5]thieno[2, 3-d]pyrimidinone, oxoindoline-amino-isoindolinone, oxoindoline-amino-benzo[b]thiophene-3-carboxylate and 1- (morpholino (pyridine) methyl)-3-(thiazolo-imino) indolinone. They have proved potent anticancer activities [22,23,24,25,26,27,28,29,30,31,32,33,55].

**(II)** These compounds **(17**, **10**, **9**, **16**, **15**, **7**, **13**, **6** and **5)** produce the highest anticancer activity due to the presence of many functional groups linked heterocyclic moieties such as imidazole; quinoxaline; oxoindoline; thiazole, pyrimidine; hydrazine-1-carboxamide; spiroindolinone; 1,3,4- thiadiazole; thioxospiroindolinone; 1,2,4- triazolidinone; pyridine; morpholine; thiophene; amino, phenyl, ethyl, and methyl groups; isatin; imidazoquinox- aline; thiazolopyrimidine; benzothienopyrimidine; and heteroatoms such as nitrogen, oxygen and sulfur. Therefore, the presence of these groups leads to the electron withdrawing; lipophilic groups at different positions on the pyridine, morpholine, thiophene and phenyl rings; and hydrophobic reactions of the quinoxaline ring in the protein active site.

**(III)** Isatin derivatives are used for their anti-proliferative and antitumor properties and are considered are drugs. They deserve special attention, due to the following reasons:

- Isatin derivatives are used as free ligands or coordinated bonds with many metal and organic ions. Hence, they provide promising anti-proliferative properties against different cancer cells.
- Isatin-containing ligands have been fused with drug delivery systems for anti-tu- mor application.
- Isatin and its derivatives have the ability to bind to nucleic acid (DNA) and generate different types of ions that act as antioxidants and also inhibit selected proteins.

**(IV)** Isatin (1*H*-indol-2, 3-Dione) is extracted as a red-orange powder from plants of the Isatis genus (Isatis tinctoria) and is a natural compound of alkaloids. Furthermore, indolic compounds are natural compounds found in these plants that have wide-ranging biological activity as anti-inflammatory and anti-tumor.

- In addition, isatin has been detected in the human body as a metabolite of tryptophan or epinephrine, and it is widely distributed in the central nervous system (CNS) [55].

## 3. Experimental Section

Through cooperation with the teamwork of researchers, a research plan was made for the synthesis of new heterocyclic compounds. These new compounds were planned to study their biological activity as anticancer, and this plan was successfully implemented.

### 3.1. General Information

All melting points were taken on an Electrothermal IA 9100 series digital melting point apparatus (Shimadzu, Tokyo, Japan). Elemental analyses were performed on Vario EL (Elementar, Langenselbold, Germany). Microanalytical data were processed in the microanalytical center of the Faculty of Science at Cairo University and National Research Centre. The IR spectra (KBr disc) were recorded using a Perkin–Elmer 1650 spectrometer (Waltham, MA, USA). NMR spectra were determined using JEOL 270 MHz and JEOL JMS-AX 500 MHz (JEOL, Tokyo, Japan) spectrometers with Me4Si as an internal standard. Mass spectra were recorded on an EI Ms-QP 1000 EX instrument (Shimadzu, Tokyo, Japan) at 70 eV. Biological evaluations were done by the anticancer unit of Mansoura University, Faculty of Pharmacy (Department of Pharmacognosy), 35516, Egypt. All starting materials and solvents were purchased from Sigma–Aldrich (Saint Louis, MO, USA).

### 3.2. Synthesis of 1-(Morpholino (Pyridin-4-yl) Methyl) Indoline-2,3-Dione ***(2)***

General procedure for the preparation of Mannich bases [51,52,53].

To a warm solution of isatin **1** (1.47 g, 0.01 mol) in sodium ethoxide solution (40 mL of ethanol and 0.23 g, 0.01 mol of sodium metal) was added the freshly distilled secondary aliphatic amine, as morpholine (0.87 mL, 0.01 mol) and isonicotinaldehyde (1.07 g, 0.01 mol) were stirred under reflux in sodium ethoxide solution (35 mL) for 8–10 h (TLC control). The reaction mixture was cooled at room temperature, poured into cold water, acidified via conc. HCl and neutralized. The formed precipitate was filtered off, dried and recrystallized from dioxane, forming yellowish crystals (85%), with m. p. = 283–285 °C (dec.). IR (*ν*, cm^−1^) KBr: 3055 (CH, aryl), 2940 (CH, aliph), 1735 (CO, keton), 1680 (CO, amide); ^1^H NMR (DMSO-*d*6, ppm) *δ* 2.60–3.05 (m, 8H, morpholine), 6.72 (s,1H, methine proton), 7.10–7.40 (m, 4H, phenyl), 7.50–7.60 (dd, 2H, *J* = 7.20 Hz, pyridine), 7.70–7.80 (dd, 2H, *J* = 7.15 Hz, pyridine); ^13^C NMR (DMSO-*d*_6_) *δ* 51.2, 58.8 (4C, 4CH_2_, morpholine), 95.4 (1C, CH, methine proton), 116.5, 117.1, 124.7, 127.2, 129.8, 135.3, 145.5, 148.6, 149.5 (11C, Ar-C), 161.2, 172.8 (2C, two carbonyl groups); MS (70 ev, %): *m/z* = 323 (M^+^, 100%); Anal. Calc. (Found) for C_18_H_17_N_3_O_3_ (323.35): C, 66.86 (66.80); H, 5.30 (5.37); N, 13.00 (13.08).

### 3.3. Synthesis of 3-((4-Bromophenyl) Imino)-1-(Morpholino (Pyridin-4-yl) Methyl) Indolin-2-One ***(3)***

A mixture of **2** (3.23 g, 0.01 mol) and 4-bromoaniline (1.72 g, 0.01 mol) in methanolic potassium hydroxide solution (prepared by dissolving 10 mmol of KOH in 40 mL methanol) was refluxed for 10–12 h. The solid precipitate was obtained by filtering, washed with 100 mL of cold water and ethanol, dried and recrystallized from acetone, forming brownish crystals (80%), with m. p. > 350 °C (dec.). IR (*ν*, cm^−1^) KBr: 3060 (CH, aryl), 2935 (CH, aliph), 1688 (CO, amide); ^1^H NMR (DMSO-*d*6, ppm) *δ* 2.65–3.10 (m, 8H, morpholine), 6.70 (s,1H, methine proton), 7.01–7.04 (dd, 2H, *J* = 7.02 Hz, 4-bromophenyl), 7.14–7.40 (m, 4H, phenyl), 7.55–7.65 (dd, 2H, *J* = 7.05 Hz, pyridine), 7.75 -7.85 (dd, 2H, *J* = 7.08 Hz, pyridine), 7.90–7.95 (dd, 2H, *J* = 7.04 Hz, 4-bromophenyl); ^13^C NMR (DMSO-*d*_6_) *δ* 51.5, 58.9 (4C, 4CH_2_, morpholine), 94.7 (1C, CH, methine proton), 116.1, 117.5, 120.8, 122.3, 124.5, 124.8, 129.1, 130.4, 132.5, 145.8, 147.7, 149.2, 151.6, 160.9 (18C, Ar-C), 164.1 (1C, carbonyl groups); MS (70 ev, %): *m/z* = 477 (M^+^, 100%); Anal. Calc. (Found) for C_24_H_21_BrN_4_O_2_ (477.36): C, 60.39 (60.30); H, 4.43 (4.50); N, 11.74 (11.66).

### 3.4. Synthesis of 2-((1-(Morpholino (Pyridin-4-yl) Methyl)-2-Oxoindolin-3-Ylidene) Amino) Benzoic Acid ***(4)***

A mixture of **2** (3.23 g, 0.01 mol) and 2-aminobenzoic acid (1.37 g, 0.01 mol) in dimethylformamide (45 mL) in the presence of anhydrous potassium carbonate (0.015 mol) was refluxed for 7–9 h with (TLC). The reaction mixture was stirred at room temperature for two hours and poured into cold water with neutralized. The solid precipitate formed was filtered off, dried and crystallized from methanol in (84%) yield, forming yellow crystals, with m. p. = 345–347 °C (dec.). IR (*ν*, cm^−1^) KBr: 3750 (br., OH), 3070 (CH, aryl), 2948 (CH, aliph), 1740 (CO, acid), 1688 (CO, amide), 1630 (C=N) and 1590 (C=C); ^1^H NMR (DMSO-*d*6, ppm) *δ* 2.70–3.15 (m, 8H, morpholine), 6.75 (s,1H, methine proton), 7.35–7.45 (dd, 2H, *J* = 7.01 Hz, pyridine), 7.50–7.88 (m, 8H, phenyl), 7.90–7.99 (dd, 2H, *J* = 7.03 Hz, pyridine), 12.60 (br., 1H, OH, D_2_O exchangeable); ^13^C NMR (DMSO-*d*_6_) *δ* 52.2, 59.1 (4C, 4CH_2_, morpholine), 95.3 (1C, CH, methine proton), 116.5, 116.8, 117.9, 122.1, 122.7, 124.4, 125.6, 128.5, 129.3, 130.7, 133.3, 146.1, 147.2, 149.6, 152.1, 161.3 (18C, Ar-C), 164.7, 167.1 (2C, two carbonyl groups); MS (70 ev, %): *m/z* = 442 (M^+^, 100%); Anal. Calc. (Found) for C_25_H_22_N_4_O_4_ (442.47): C, 67.86 (67.80); H, 5.01 (5.08); N, 12.66 (12.73).

### 3.5. Synthesis of 1-(Morpholino (Pyridin-4-yl) Methyl) -3-(Thiazol-2-Ylimino) Indolin-2-One ***(5)***

A solution of **2** (3.23 g, 0.01 mol), 2-aminothiazole (1.00 g, 0.01 mol) and anhydrous potassium carbonate (1.4 g, 0.01 mol) in dry dimethylformamide (40 mL) was used as a solvent. The reaction mixture was heated and refluxed for 9–11 h under control (TLC), and then allowed to cool and poured into cold water. The final precipitate was filtered and recrystallized from dioxane in (82%) yield, forming yellowish crystals, with m. p. = 333–335 °C (dec.). IR (*ν*, cm^−1^) KBr: 3061 (CH, aryl), 2957 (CH, aliph), 1678 (CO, amide) and 1632 (C=N), 1585 (C=C). ^1^H NMR (DMSO-*d*6, ppm) *δ* 2.72–3.18 (m, 8H, morpholine), 6.67 (s,1H, methine proton), 7.30–7.40 (dd, 2H, *J* = 7.01 Hz, pyridine), 7.44–7.72 (m, 4H, phenyl), 7.75–7.80 (d, 1H, *J* = 6.90 Hz, thiazole), 7.82–7.87 (d, 1H, *J* = 6.92 Hz, thiazole), 7.89–7.98 (dd, 2H, *J* = 7.03 Hz, pyridine); ^13^C NMR (DMSO-*d*_6_) *δ* 52.5, 59.7 (4C, 4CH_2_, morpholine), 94.4 (1C, CH, methine proton), 116.4, 117.2, 117.6, 123.5, 124.1, 129.7, 130.5, 141.1, 146.3, 147.6, 149.3, 161.5, 165.9 (15C, Ar-C), 168.2 (1C, carbonyl groups); MS (70 ev, %): *m/z* = 405 (M^+^, 100%); Anal. Calc. (Found) for C_21_H_19_N_5_O_2_S (405.48): C, 62.21 (62.30); H, 4.72 (4.80); N, 17.27 (17.18).

### 3.6. Synthesis of Ethyl -2-((1-(Morpholino (Pyridin-4-yl) Methyl)-2-Oxoindolin-3-Ylidene) amino)-4,5,6,7-Tetrahydrobenzo[b]thiophene-3-Carboxylate ***(6)***

To a solution of compound **2** (3.23 g, 0.01 mol), ethyl 2-amino-4,5,6,7-tetrahy- drobenzo[b]thiophene-3-carboxylate (2.25 g, 0.01 mol) and anhydrous potassium carbonate (1.4 g, 0.01 mol) were added in dry dimethylformamide (40 mL), and the reaction mixture was refluxed for 10–12 h with control by (TLC). After cooling at room temperature, the reaction solution was poured onto ice water. The product precipitate was filtered off, dried and recrystallized from toluene in (81%) yield, forming yellow crystals, with m. p. > 350 °C (dec.). IR (*ν*, cm^−1^) KBr: 3070 (CH, aryl), 2960 (CH, aliph), 1775 (CO, ester), 1682 (CO, amide) and 1631 (C=N), 1590 (C=C); ^1^HNMR (DMSO-*d_6_*, ppm) *δ* 1.08–1.15 (m,8H,cyclohexane), 1.28–1.35 (t, 3H, *J* =7.20 Hz, CH_3_), 2.75–3.22 (m, 8H, morpholine), 4.42–4.49 (q, 2H, *J* = 7.22 Hz, CH_2_), 6.62 (s,1H, methine proton), 7.25–7.33 (dd, 2H, *J* = 7.09 Hz, pyridine), 7.55–7.82 (m, 4H, phenyl), 7.90–7.95 (dd, 2H, *J* = 7.08 Hz, pyridine); ^13^C NMR (DMSO-*d_6_*) *δ* 18.8 (1C, CH_3_), 21.5, 22.4, 23.1, 24.7 (4C, 4CH_2_, cyclohexane), 52.8, 58.9 (4C, 4CH_2_, morpholine), 61.2 (1C,CH_2_), 94.7 (1C, CH, methine proton), 116.3, 117.9, 123.7, 124.5, 127.1, 128.8, 129.5, 130.9, 138.6, 146.1, 147.7, 149.5, 151.3, 160.8 (16C, Ar-C), 164.2, 166.9 (2C, carbonyl groups); MS (70 ev, %): *m/z* = 530 (M^+^, 100%); Anal. Calc. (Found) for C_29_H_30_N_4_O_4_S (530.64): C, 65.64 (65.71); H, 5.70 (5.62); N, 10.56 (10.50).

### 3.7. Synthesis of 1-(1-(Morpholino(Pyridin-4-yl)Methyl)-2-Oxoindolin-3-yl)-5,6,7,8-Tetrahydro benzo[4,5]thieno[2,3-d]pyrimidin-4(1H)-One ***(7)***

A mix of compound **6** (5.30 g, 0.01 mol) and formamide (0.5 mL, 0.01 mol) was stirred under reflux in dimethylformamide (35 mL) in the presence of anhydrous potassium carbonate (0.015 mol) for 16–18 h under control (TLC). The reaction solution was cooled at room temperature, poured into cold water and neutralized. The solid product formed was filtered off, dried and crystallized from dioxane in (80%) yield, forming yellowish crystals, with m. p. > 350 °C (dec.). IR (*ν*, cm^−1^) KBr: 3075 (CH, aryl), 2970 (CH, aliph), 1690, 1685 (2CO, amide) and 1634 (C=N), 1588 (C=C); ^1^HNMR (DMSO-*d_6_*, ppm) *δ* 110–1.18 (m,8H,cyclohexane), 2.80–3.27 (m, 8H, morpholine), 5.10 (s,1H, CH, pyrrole), 6.58 (s,1H, methine proton), 7.35–7.44 (dd, 2H, *J* = 7.04 Hz, pyridine), 7.59–7.88 (m, 4H, phenyl), 7.92–7.97 (dd, 2H, *J* = 7.05 Hz, pyridine), 8.15 (s,1H, CH, pyrimidine); ^13^C NMR (DMSO-*d_6_*) *δ* 21.7, 22.8, 23.4, 24.9 (4C, 4CH_2_, cyclohexane), 52.9, 59.1 (4C, 4CH_2_, morpholine), 77.4 (1C, CH_,_ pyrrole), 93.9 (1C, CH, methine proton), 117.7, 124.1, 124.6, 125.8, 127.5, 127.9, 129.7, 135.8, 138.8, 139.6, 144.5, 146.3, 148.2, 149.7, (16C, Ar-C), 163.8, 167.1 (2C, carbonyl groups); MS (70 ev, %): *m/z* = 513 (M^+^, 95%); Anal. Calc. (Found) for C_28_H_27_N_5_O_3_S (513.62): C, 65.48 (65.40); H, 5.30 (5.37); N, 13.64 (13.57).

### 3.8. Synthesis of Ethyl -2-(1-(Morpholino (Pyridin-4-yl) Methyl)-2-Oxoindolin-3-Ylidene) Hydrazine-1-Carboxylate ***(8)***

A mix of compound **2** (3.23 g, 0.01 mol), ethyl hydrazinecarboxylate (1.04 g, 0.01 mol) and anhydrous potassium carbonate (1.4 g, 0.01 mol) was formed in dry dimethylformamide (45 mL). The reaction mixture was heated under reflux for 11–13 h under control (TLC) and then allowed to cool and poured into cold water. The precipitate was filtered off, dried and recrystallized from n-hexane in (84%) yield, yellowish crystals, m.p = 308–310 °C (dec.). IR (*ν*, cm^−1^) KBr: 3325 (br., NH), 3055 (CH, aryl), 2960 (CH, aliph), 1691, 1684 (2CO, amide), 1628 (C=N) and 1582 (C=C); ^1^HNMR (DMSO-*d*6, ppm) *δ* 1.29–1.36 (t,3H, *J* = 7.12 Hz, CH_3_), 2.72–3.19 (m, 8H, morpholine), 4.35–4.42 (q, 2H, *J* = 7.10 Hz, CH_2_), 6.55 (s,1H, methine proton), 7.32–7.41 (dd, 2H, *J* = 7.01 Hz, pyridine), 7.52–7.81 (m, 4H, phenyl), 7.88–7.95 (dd, 2H, *J* = 7.02 Hz, pyridine), 10.50 (br., 1H, NH, D_2_O exchangeable); ^13^C NMR (DMSO-*d*_6_) *δ* 19.1 (1C, CH_3_), 52.6, 58.5 (4C, 4CH_2_, morpholine), 61.7 (1C,CH_2_), 93.5 (1C, CH, methine proton), 116.1, 117.5, 124.1, 124.6, 129.2, 131.8, 132.7, 146.3, 147.5, 149.6 (12C, Ar-C), 161.1, 164.3 (2C, carbonyl groups); MS (70 ev, %): *m/z* = 409 (M^+^, 100%); Anal. Calc. (Found) for C_21_H_23_N_5_O_4_ (409.45): C, 61.60 (61.67); H, 5.66 (5.60); N, 17.10 (17.17).

### 3.9. Synthesis of N-(2-Mercapto-6-oxo-1,6-Dihydropyrimidin-4-yl)-2-(1-(Morpholino (Pyridin-4- yl) methyl)-2-Oxoindolin-3-Ylidene) Hydrazine-1-Carboxamide ***(9)***

A suspension of compound 4 (4.09 g, 0.01 mol) and 6-amino-2-mercaptopyrimidin- 4(3*H*)-one (1.43 g, 0.01 mol) in dry dimethylformamide (40 mL) containing a catalytic amount of TEA (0.5 mL) was stirred and heated under reflux for 13–15 h with (TLC) control. The reaction solution was cooled; the solid precipitate was filtered off, dried and recrystallized from methanol/ DMF in (78%) yield, forming brownish crystals, with m. p. > 350 °C (dec.). IR (*ν*, cm^−1^) KBr: 3330–3270 (br., 3NH), 3060 (CH, aryl), 2970 (CH, aliph), 1695, 1688, 1682 (3CO, amide), 1631 (C=N) and 1585 (C=C); ^1^HNMR (DMSO-*d*6, ppm) *δ* 2.66 (s, 1H, SH), 2.75–3.25 (m, 8H, morpholine), 6.50 (s,1H, methine proton), 6.85 (s, 1H, CH, pyrimidine), 7.36–7.42 (dd, 2H, *J* = 7.06 Hz, pyridine), 7.50–7.80 (m, 4H, phenyl), 7.85–7.92 (dd, 2H, *J* = 7.04 Hz, pyridine), 9.90 (br., 1H, NH, D_2_O exchangeable), 10.80 (br., 1H, NH, D_2_O exchangeable), 11.70 (br., 1H, NH, D_2_O exchangeable); ^13^C NMR (DMSO-*d*_6_) *δ* 51.9, 58.7 (4C, 4CH_2_, morpholine), 81.5 (1C, CH, pyrimidine), 94.2 (1C, CH, methine proton), 116.3, 117.6, 124.4, 124.8, 129.6, 131.5, 132.1, 146.7, 147.8, 149.7, 153.1, 160.2 (14C, Ar-C), 161.5, 164.1, 166.2 (3C, three carbonyl groups); MS (70 ev, %): *m/z* = 409 (M^+^, 100%); Anal. Calc. (Found) for C_23_H_22_N_8_O_4_S (506.54): C, 54.54 (54.62); H, 4.38 (4.31); N, 22.12 (22.07).

### 3.10. Synthesis of N-(3,5-Dioxo-2,3-Dihydro-5H-Thiazolo [3,2-a] Pyrimidin-7-yl)-2-(1- (Morphol- ino (Pyridin-4-yl) Methyl)-2-Oxoindolin-3-Ylidene) Hydrazine-1-Carboxamide ***(10)***

**Method A:** A mixture of compound **9** (5.06 g, 0.01 mol), chloroacetic acid (0.94 g, 0.01 mol), and 0.02 mol of anhydrous sodium acetate was stirred under reflux in (40 mL) of glacial acetic acid and (25 mL) of acetic anhydride in a water bath (85–95 °C) for 17–19 h under control (TLC). The reaction mixture was cooled at room temperature and poured into cold water (100 mL). The solid precipitate was filtered off, dried and crystallized from a suitable solvent to yield **(10)**. **Method B:** A solution of **9** (5.06 g, 0.01 mol) and chloroacetic acid (0.94 g, 0.01 mol) in dimethylformamide (35 mL) was heated and refluxed for 14–16 h under control (TLC). The final precipitate was filtered off, dried and crystallized from DMF/methanol in good yields (75%), forming yellowish crystals, with m. p. > 350 °C (dec.). IR (*ν*, cm^−1^) KBr: 3325–3280 (br., 2NH), 3055 (CH, aryl), 2965(CH, aliph), 1691, 1686, 1681, 1675 (4CO, amide), 1634 (C=N) and 1582 (C=C); ^1^HNMR (DMSO-*d*6, ppm) *δ* 2.78–3.28 (m, 8H, morpholine), 4.22 (s, 2H, CH_2,_ thiazole), 6.58 (s,1H, methine proton), 6.88 (s, 1H, CH, pyrimidine), 7.39–7.45 (dd, 2H, *J* = 7.08 Hz, pyridine), 7.58–7.87 (m, 4H, phenyl), 7.91–7.98 (dd, 2H, *J* = 7.07 Hz, pyridine),10.10 (br., 1H, NH, D_2_O exchangeable), 11.60 (br., 1H, NH, D_2_O exchangeable); ^13^C NMR (DMSO-*d*_6_) *δ* 32.1 (1C, CH_2,_ thiazole), 52.3, 59.1 (4C, 4CH_2_, morpholine), 82.3 (1C, CH, pyrimidine), 93.5 (1C, CH, methine proton), 116.1, 117.9, 124.5, 124.7, 129.5, 131.4, 132.5, 146.2, 147.6, 149.5, 153.3, 159.1 (14C, Ar-C), 161.8, 163.5, 165.7, 169.1 (4C, four carbonyl groups); MS (70 ev, %): *m/z* = 546 (M^+^, 92%); Anal. Calc. (Found) for C_25_H_22_N_8_O_5_S (546.56): C, 54.94 (54.86); H, 4.06 (4.01); N, 20.50 (20.59).

### 3.11. Synthesis of 3-((2-Aminophenyl) Amino)-3-Hydroxy-1-(Morpholino (Pyridin-4-yl) Methyl) Indolin-2-One ***(11)***

To a mixture of **2** (3.23 g, 0.01 mol), benzene-1,2-diamine (1.08 g, 0.01 mol) in absolute methanol (30 mL) and diethylamine (0.73 mL, 0.01 mol) was added. The reaction solution was stirred and refluxed at room temperature for ~8–10 h and monitored by using TLC. The solid formed was filtered off, washed with water, dried and recrystallized from DMF in good yields (83%), forming yellow crystals, with m. p.= 297–300 °C (dec.). IR (*ν*, cm^−1^) KBr: 3415 (br., NH_2_), 3370–3330 (brs., OH, NH), 3060 (CH, aryl), 2950 (CH, aliph), 1682 (CO, amide), 1630 (C=N) and 1580 (C=C); ^1^H NMR (DMSO-*d*6, ppm) *δ* 2.70–3.10 (m, 8H, morpholine), 6.25 (br., 2H, NH_2_, D_2_O exchangeable), 6.60 (s,1H, methine proton), 7.22–7.30 (dd, 2H, *J* = 7.09 Hz, pyridine), 7.35–7.80 (m, 8H, phenyl), 7.85–7.97(dd, 2H, *J* = 7.07 Hz, pyridine), 11.10 (br., 1H, NH, D_2_O exchangeable), 12.60 (br., 1H, OH, D_2_O exchangeable);^13^C NMR (DMSO-*d*_6_) *δ* 51.6, 58.5 (4C, 4CH_2_, morpholine), 93.1 (1C, CH, methine proton), 111.4, 112.1, 114.8, 116.9, 118.2, 120.3, 124.4, 128.1, 128.9, 133.3, 135.2, 135.7, 137.5, 145.2, 147.4, 149.7 (18C, Ar-C), 165.7 (1C, carbonyl group); MS (70 ev, %): *m/z* = 431 (M^+^, 90%); Anal. Calc. (Found) for C_24_H_25_N_5_O_3_ (431.50): C, 66.81 (66.73); H, 5.84 (5.75); N, 16.23 (16.32).

### 3.12. Synthesis of 3-((2-Aminophenyl) Imino)-1-(Morpholino (Pyridin-4-yl) Methyl) Indolin-2-one ***(12)***

A solution of compound **11** (4.31 g, 0.01 mol) in glacial acetic acid (30 mL) and concentrated HCl (0.5 mL) was heated and refluxed at 80–90 °C for ~1–2 h. After cooling, the reaction mixture was cooled and poured into ice water. The solid precipitate that formed was filtered off, washed with water, air-dried and crystallized from dioxane in good yields (80%), forming yellowish crystals, with m. p. = 320–322 °C (dec.). IR (*ν*, cm^−1^) KBr: 3410 (br., NH_2_), 3057 (CH, aryl), 2953 (CH, aliph), 1684 (CO, amide), 1637 (C=N) and 1583 (C=C); ^1^H NMR (DMSO-*d*6, ppm) *δ* 2.77–3.16 (m, 8H, morpholine), 6.35 (br., 2H, NH_2_, D_2_O exchangeable), 6.65 (s,1H, methine proton), 7.28–7.37 (dd, 2H, *J* = 7.10 Hz, pyridine), 7.40–7.85 (m, 8H, phenyl), 7.87–7.99 (dd, 2H, *J* = 7.12 Hz, pyridine); ^13^C NMR (DMSO-*d*_6_) *δ* 51.8, 58.7 (4C, 4CH_2_, morpholine), 93.5 (1C, CH, methine proton), 115.9, 117.4, 117.8, 123.7, 124.1, 124.6, 127.2, 128.4, 129.6, 132.3, 140.8, 142.5, 146.3, 147.1, 149.5, 161.7 (18C, Ar-C), 165.9 (1C, carbonyl group); MS (70 ev, %): *m/z* = 413 (M^+^, 100%); Anal. Calc. (Found) for C_24_H_23_N_5_O_2_ (413.48): C, 69.72 (69.80); H, 5.61 (5.55); N, 16.94 (16.88).

### 3.13. Synthesis of 2-(2-((1-(Morpholino (Pyridin-4-yl) Methyl)-2-Oxoindolin-3-Ylidene) Amino) phenyl) Isoindoline-1,3-Dione ***(13)***

A solution of compound **12** (4.13 g, 0.01 mol) and phthalic anhydride (1.48 g, 0.01 mol) in absolute methanol (45 mL) containing a few drops of glacial acetic acid was stirred and heated under reflux for ~10–12 h with (TLC control). The solid precipitate obtained was filtered, dried and recrystallized from DMF in good yields (75%), forming white crystals, with m. p. > 350 °C (dec.). IR (*ν*, cm^−1^) KBr: 3062 (CH, aryl), 2958(CH, aliph), 1688, 1682, 1677 (3CO, amide), 1633(C=N) and 1585 (C=C); ^1^H NMR (DMSO-*d*6, ppm) *δ* 2.80–3.20 (m, 8H, morpholine), 6.60 (s,1H, methine proton), 7.13–7.22 (dd, 2H, *J* = 7.13 Hz, pyridine), 7.31–7.72 (m, 8H, phenyl), 7.75–7.88 (dd, 2H, *J* = 7.11 Hz, pyridine), 7.91–7.99 (m, 4H, phenyl); ^13^C NMR (DMSO-*d*_6_) *δ* 52.3, 59.1 (4C, 4CH_2_, morpholine), 93.8 (1C, CH, methine proton), 116.2, 118.5, 122.6, 123.4, 124.3, 124.8, 126.4, 127.7, 129.4, 129.8, 131.4, 131.9, 132.4, 132.7, 146.2, 146.8, 147.6, 149.7, 161.9 (24C, Ar-C), 166.1, 168.5 (3C, three carbonyl groups); MS (70 ev, %): *m/z* = 543 (M^+^, 100%); Anal. Calc. (Found) for C_32_H_25_N_5_O_4_ (543.58): C, 70.71 (70.78); H, 4.64 (4.57); N, 12.88 (12.80).

### 3.14. Synthesis of 2-(1-(Morpholino (Pyridin-4-yl) Methyl)-2-Oxoindolin-3-Ylidene) Hydrazine-1-Carbothioamide ***(14)***

To a warm mixture of thiosemicarbazide (0.91 g, 0.01 mol) in glacial acetic acid (30 mL), 1-(morpholino (pyridine) methyl) indoline-2,3-dione **2** (3.23 g, 0.01 mol) was added. The reaction mixture was heated under reflux for ~5–7 h. The formed solid precipitate was filtered off, washed with ethanol / water and dried, then recrystallized from DMF to obtain yellow crystals in good yield (84%), with m. p. > 350 °C (dec.). IR (*ν*, cm^−1^) KBr: 3430, 3320 (NH_2_ and NH), 3052 (CH, aryl), 2943 (CH, aliph), 1678 (C=O), 1629 (C=N), 1582 (C=C), and 1475 (C=S); ^1^H NMR (DMSO-*d*6, ppm) *δ* 2.72–3.13 (m, 8H, morpholine), 6.61 (s,1H, methine proton), 6.90 (br., 2H, NH_2_, D_2_O exchangeable), 7.22–7.50 (m, 4H, phenyl), 7.61–7.70 (dd, 2H, *J* = 7.17 Hz, pyridine), 7.77–7.90 (dd, 2H, *J* = 7.14 Hz, pyridine), 11.35 (br., 1H, NH, D_2_O exchangeable); ^13^C NMR (DMSO-*d*_6_) *δ* 51.4, 59.9 (4C, 4CH_2_, morpholine), 94.5 (1C, CH, methine proton), 116.9, 118.3, 124.1, 124.6, 129.2, 131.5, 132.1, 145.9, 147.1, 149.6 (12C, Ar-C), 165.1 (1C, carbonyl group), 178.8 (1C,C=S); MS (70 ev, %): *m/z* = 396 (M^+^, 100%); Anal. Calc. (Found) for C_19_H_20_N_6_O_2_S (396.47): C, 57.56 (57.65); H, 5.08 (5.02); N, 21.20 (21.14).

### 3.15. Synthesis of 1- (Morpholino (Pyridin-4-yl) Methyl)-5′-Thioxospiro [Indoline-3,3′-[1,2,4] Triazolidin]-2-One ***(15)***

**Method A:** Compound **14** (3.96 g, 0.01 mol) was stirred under reflux in dry chloroform (35 mL) or glacial acetic acid (30 mL), and triethylamine (1 mL) was added, as a catalyst for a long time (~15–17 h) under TLC control. The mixture was evaporated under reduced pressure. The precipitate produced was washed many times with methanol and crystallized form dioxane to produce **(15)** in high yields. **Method B:** This was a one-pot synthesis method. A mixture of thiosemicarbazide (0.91 g, 0.01 mol) in glacial acetic acid (40 mL) and compound **2** (3.23 g, 0.01 mol) was formed. The reaction solution was heated and under reflux for a long time (~19–21 h) under control (TLC). The final precipitate was filtered off, washed with ethanol/water and dried, then recrystallized from dioxane to obtain yellowish crystals in good yield (78%), with m. p. > 350 °C (dec.). IR (*ν*, cm^−1^) KBr: 3350–3230 (3NH), 3060 (CH, aryl), 2950 (CH, aliph), 1688 (C=O), 1633 (C=N), 1585 (C=C), and 1450 (C=S); ^1^H NMR (DMSO-*d*6, ppm) *δ* 2.70–3.11 (m, 8H, morpholine), 6.64 (s,1H, methine proton), 7.15–7.44 (m, 4H, phenyl), 7.55–7.65 (dd, 2H, *J* = 7.13 Hz, pyridine), 7.80–7.95 (dd, 2H, *J* = 7.16 Hz, pyridine), 10.10 (br., 1H, NH, D_2_O exchangeable), 10.40 (br., 1H, NH, D_2_O exchangeable), 11.55 (br., 1H, NH, D_2_O exchangeable); ^13^C NMR (DMSO-*d*_6_) *δ* 51.8, 60.2 (4C, 4CH_2_, morpholine), 93.7 (1C, CH, methine proton), 97.1 (1C, 5′-thioxospiro[indoline- 3,3′-[1,2,4]triazolidin]-2-one), 117.8, 123.1, 124.5, 128.3, 130.6, 136.8, 145.4, 147.5, 149.5 (11C, Ar-C), 168.2 (1C, carbonyl group), 180.4 (1C,C=S); MS (70 ev, %): *m/z* = 396 (M^+^, 94%); Anal. Calc. (Found) for C_19_H_20_N_6_O_2_S (396.47): C, 57.56 (57.48); H, 5.08 (5.14); N, 21.20 (21.27).

### 3.16. Synthesis of 5′-Amino-1-(Morpholino (Pyridin-4-yl) Methyl) -3′H-Spiro [Indoline-3,2′-[1,3,4] Thiadiazol]-2-One ***(16)***

A mixture of compound **14** (3.96 g, 0.01 mol), iodine (1.26 g, 0.01 mol) and anhydrous potassium carbonate (1.4 g, 0.01 mol) was stirred under reflux in dry 1,4-dioxane (40 mL) or dimethylformamide (40 mL), for ~13–15 h under TLC control. The solution was evaporated under reduced pressure. After drying, the precipitate produced was washed with 5% Na_2_S_2_O_3_ (30 mL) and extracted from mixture of CH_2_Cl_2_ / MeOH (10:1, 10 mL × 4). The final precipitate was dried over anhydrous sodium sulphate and was purified using petroleum ether/ethyl-acetate mixture as eluent by silica gel column chromatography, yielding the synthesis of conforming analogs **(16)**. **Method B:** A solution of **14** (3.96 g, 0.01 mol) and triethylamine (3 drops) in ethanol (30 mL) was heated under reflux for 6–8 h with TLC control. After cooling, the solid precipitate was collected and crystallized from dioxane to produce yellow crystals in good yield (76%), with m. p. > 350 °C (melted). IR (*ν*, cm^−1^) KBr: 3420, 3335 (NH_2_ and NH), 3066 (CH, aryl), 2957 (CH, aliph), 1682 (C=O), 1631 (C=N), 1583 (C=C); ^1^H NMR (DMSO-*d*6, ppm) *δ* 2.68–3.10 (m, 8H, morpholine), 6.60 (s,1H, methine proton), 6.85 (br., 2H, NH_2_, D_2_O exchangeable), 7.22–7.51 (m, 4H, phenyl), 7.61–7.75 (dd, 2H, *J* = 7.18 Hz, pyridine), 7.78–7.93 (dd, 2H, *J* = 7.17 Hz, pyridine), 10.25 (br., 1H, NH, D_2_O exchangeable); ^13^C NMR (DMSO-*d*_6_) *δ* 52.4, 61.7 (4C, 4CH_2_, morpholine), 78.5 (1C, 3′*H*-spiro[indoline-3,2′-[1,3,4]thiadiazol]-2-one), 91.6 (1C, CH, methine proton), 118.2, 124.3, 124.7, 127.9, 128.5, 130.4, 145.1, 146.7, 149.3, 149.9 (12C, Ar-C), 167.5 (1C, carbonyl group); MS (70 ev, %): *m/z* = 396 (M^+^, 90%); Anal. Calc. (Found) for C_19_H_20_N_6_O_2_S (396.47): C, 57.56 (57.50); H, 5.08 (5.16); N, 21.20 (21.30).

### 3.17. Synthesis of 1-(Morpholino (Pyridin-4-yl) Methyl)-3-((2-Thioxo-2,3-Dihydro-1H- Imidazo [4,5-b] Quinoxalin-1-yl) Imino) Indolin-2-One ***(17)***

A mixture of compound **14** (3.96 g, 0.01 mol) and 2, 3-dichloroquinoxaline (1.99 g, 0.01 mol) in absolute ethanol containing (0.2 mL) of triethylamine as a catalyst was refluxed for 22–25 h under control (TLC). The reaction solution was cooled and the deposited solid was filtered off, dried and recrystallized from dimethylformamide to produce yellowish crystals in good yield (74%), with m. p. > 350 °C (melted). IR (*ν*, cm^−1^) KBr: 3340 (NH), 3075 (CH, aryl), 2970 (CH, aliph), 1686 (C=O), 1634 (C=N), 1587 (C=C), 1480 (C=S); ^1^H NMR (DMSO-*d*6, ppm) *δ* 2.75–3.18 (m, 8H, morpholine), 6.55 (s,1H, methine proton), 7.05–7.67 (m, 8H, phenyl), 7.70–7.80 (dd, 2H, *J* = 7.05 Hz, pyridine), 7.85–7.98 (dd, 2H, *J* = 7.07 Hz, pyridine), 10.60 (br., 1H, NH, D_2_O exchangeable); ^13^C NMR (DMSO-*d*_6_) *δ* 52.7, 61.9 (4C, 4CH_2_, morpholine), 92.8 (1C, CH, methine proton), 116.2, 118.5, 124.1, 124.4, 124.6, 124.8, 129.6, 130.4, 131.5, 134.2, 146.8, 147.1, 149.6, 153.4, (20 C, Ar-C), 166.2 (1C, carbonyl group), 175.1 (1C,C=S); MS (70 ev, %): *m/z* = 522 (M^+^, 97%); Anal. Calc. (Found) for C_27_H_22_N_8_O_2_S (522.59): C, 62.06 (62.12); H, 4.24 (4.18); N, 21.44 (21.52).

### 3.18. Pharmacological Screening

#### 3.18.1. Ethics Approval and Consent to Participate

No humans or animals were used in this study; nevertheless, all the procedures were carried out under the Medical Research Ethics Committee of Mansoura University, Faculty of Pharmacy, Department of Pharmacognosy, 35516, Egypt.

#### 3.18.2. Human and Animal Rights

No humans or animals were used in the study. The research was conducted according to ethical standards in vitro.

#### 3.18.3. Chemicals and Drugs

Types of human carcinoma cancer cell line (MGC-803, MCF-7, CNE2 and KB) are derived from the National Cancer Institute, Cairo University, Cairo, Egypt, and 5-Fluorouracil and DMSO were purchased from Sigma-Aldrich (Saint Louis, MO, USA).

#### 3.18.4. Materials and Methods (In Vitro Cytotoxicity)

The in vitro cytotoxicity of the synthesized compounds against different cancer cell lines was measured with the MTT assay, in agreement with the method found in [22,23,24,25,26,27,28,29,30,31,32,33,54]. The MTT assay is based on the reduction of the soluble 3-(4,5-dimethyl-2-thiazolyl)-2,5-diphenyl-2*H*-tetrazoliumbromide (MTT) into a blue-purple formazan product, mainly by mitochondrial reductase activity inside living cells. The cells used in the cytotoxicity assay were cultured in the suitable cell culture medium (RPMI 1640) supplemented with 10% fetal calf serum. Cells suspended in the medium (2Y’ 104/mL) were plated in 96-well culture plates and incubated at 37 °C in a 5% CO_2_ incubator. After 12 h, the test sample (2 mL) was added to the cells (2Y’ 104) in 96-well plates and cultured at 37 °C for 3 days. The cultured cells were mixed with 20 mL of MTT solution and incubated for 4 h at 37 °C. The supernatant was carefully removed from each well, and 100 mL of DMSO was added to each well to dissolve the formazan crystals, which were formed by the cellular reduction of MTT. After mixing with a mechanical plate mixer, the absorbance of each well was measured by a microplate reader using a test wavelength of 570 nm. The results were expressed as the IC_50_, which is the concentration of the drugs inducing a 50% inhibition of cell growth of treated cells, when compared to the growth of control cells. Each experiment was performed at least three times. There was good reproducibility between replicate wells with standard errors below 10%. Each experiment was repeated on three different days and conducted in triplicate. The relative cell anti-proliferative was measured according to the following equation: % cytotoxicity = (1 − As/Ab) × 100, where As = absorbance of each sample and Ab = absorbance of the blank. The probity analysis using the SPSS software program (version 20, SPSS Inc., Chicago, IL, USA) was used to determine each IC_50_.

## 4. Conclusions

In this article, isatin derivatives (1-(morpholino (pyridin-4-yl) methyl) indoline-2,3-dione) were converted into polyfunctionalized heterocycles with promising cytotoxic activity. Different derivatives were prepared, including 3-((bromophenyl) imino)-1- (morpholino (pyridine) methyl) indolinone, 2-((oxoindoline) amino) benzoic acid, 3-(thi- azoloimino) indolinone, ethyl-2-((oxoindoline) amino)-benzothiophene-3-carboxylate, 1- (oxoindoline)-benzothieno[2,3-d]pyrimidinone, ethyl-2-(oxoindoline) hydrazine-1-carb- oxylate, *N*-(mercapto-pyrimidine)-2-(oxoindoline) hydrazine-1-carboxamide, *N*-(thiazolo [3,2-a]pyrimidine)-2-(oxoindoline) hydrazine-1-carboxamide, 3-((amino-phenyl) amino)- 3-hydroxy-indolinone, 3-((amino-phenyl) imino)-indolinone, 2-(2-((oxoindoline) amino) phenyl) isoindolinone, 2-(oxoindoline) hydrazine-1-carbothioamide, 5′-thioxospiro[ind- oline-3, 3′-[1,2,4]triazolidin]-one, 5′-amino-spiro [indoline-3,2′-[1,3,4] thiadiazol]-one and 3-((2-thioxo-imidazo[4,5-b]quinoxaline)imino) indolinone in good yields. Isatin with imidazo [4, 5-b] quinoxaline, thiazolo [3,2-a] pyrimidine, hydrazine-1-carboxamide, spiro[indoline-3,2′-[1,3,4]thiadiazol]-2-one, 5′-thioxospiro[indoline-3,3′-[1,2,4] triazolidin]-2-one moiety and substituted heteroatoms as nitrogen, oxygen and sulfur showed potent cytotoxic activity against the different types of human carcinoma cells lines (MGC-803, MCF-7, CNE2 and KB). The simplicity of the synthetic methods and the potent activity of the synthesized compounds render these derivatives interesting targets for future development as cytotoxic agents. So, the imidazo [4,5-b] quinoxaline-imino- indolinone is a promising anticancer molecule with diverse effects, including angiopreventive and anti-proliferative effects, in addition to apoptosis-inducing effects.

## Figures and Tables

**Table 1:** Cytotoxic activities of the new isatin derivatives against diverse human cancer cell lines.

Compounds	In Vitro Cytotoxicity IC_50_ (µM) ± SD				TI
	MGC-803 ^a^	MCF-7 ^a^	CNE2 ^a^	KB ^a^	
**1**	>50	>50	>50	>50	2.1
**2**	35.7 ± 1.5	35.4 ± 1.2	35.3 ± 1.6	35.2 ± 1.4	1.9
**3**	27.8 ± 1.2	27.6 ± 1.4	27.4 ± 1.5	27.1 ± 1.2	1.8
**4**	20.9 ± 1.4	20.7 ± 1.5	20.5 ± 1.3	20.2 ± 1.1	1.7
**5**	11.8 ± 1.3	11.6 ± 1.4	11.5 ± 1.2	11.3 ± 1.3	1.4
**6**	10.9 ± 1.1	10.8 ± 1.3	10.7 ± 1.1	10.5 ± 1.5	1.3
**7**	10.5 ± 1.2	10.4 ± 1.1	10.2 ± 1.4	10.1 ± 1.2	1.1
**8**	12.9 ± 1.3	12.8 ± 1.5	12.6± 1.4	12.5 ± 1.6	1.6
**9**	9.9 ± 1.1	9.8 ± 1.2	9.7 ± 1.1	9.6 ± 1.2	0.6
**10**	9.8 ± 1.2	9.7 ± 1.1	9.6 ± 1.3	9.5 ± 1.1	0.5
**11**	14.5 ± 1.1	14.3 ± 1.3	14.2± 1.4	14.1 ± 1.2	1.4
**12**	13.8 ± 1.5	13.7 ± 1.2	13.5± 1.1	13.3 ± 1.4	1.3
**13**	10.7 ± 1.3	10.6 ± 1.5	10.4 ± 1.3	10.2 ± 1.1	1.2
**14**	12.6 ± 1.4	12.4 ± 1.6	12.3± 1.5	12.1 ± 1.2	1.5
**15**	10.3 ± 1.1	10.2 ± 1.2	10.1 ± 1.3	9.9 ± 1.4	0.9
**16**	10.1 ± 1.3	10.0 ± 1.4	9.9 ± 1.2	9.8 ± 1.3	0.8
**17**	9.7 ± 1.1	9.6 ± 1.2	9.5 ± 1.1	9.4 ± 1.2	0.4
**5-Fluorouracil**	10.7 ± 1.2	10.5 ± 1.1	10.3 ± 1.3	10.1 ± 1.1	0.7

^a^**MGC-803** cells are drug-sensitive human gastric carcinoma cells; **MCF-7** cells are drug-sensitive human breast adenocarcinoma cells; **CNE2** cells are drug-sensitive human nasopharyngeal carcinoma cells; and **KB** cells are drug-sensitive human oral carcinoma cells.

**Table 2:** The best new isatin derivatives with cytotoxic activity against different human cancer cell lines.

Chemical Structures	In vitro Cytotoxicity IC_50_ (µM) ± SD				TI
	MGC-803 ^a^	MCF-7 ^a^	CNE2 ^a^	KB ^a^	
	Functional Groups/Heterocyclic Moieties				
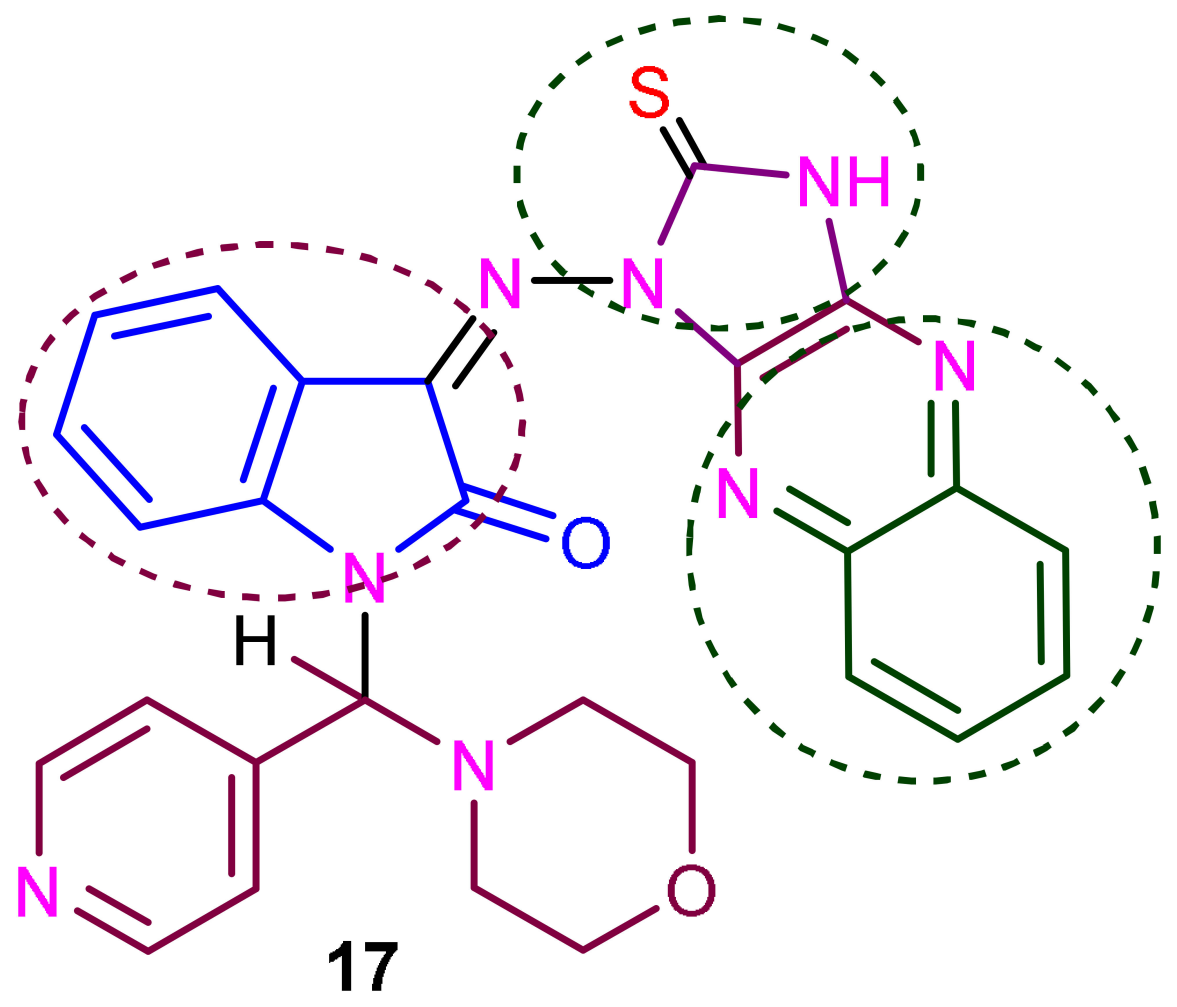	9.7 ± 1.1	9.6 ± 1.2	9.5 ± 1.1	9.4 ± 1.2	0.4
	Isatin, morpholine, pyridine, methyl, thioxo, imidazo [4,5-b] quinoxaline, carbonyl group, nitrogen, oxygen and sulfur atoms.				
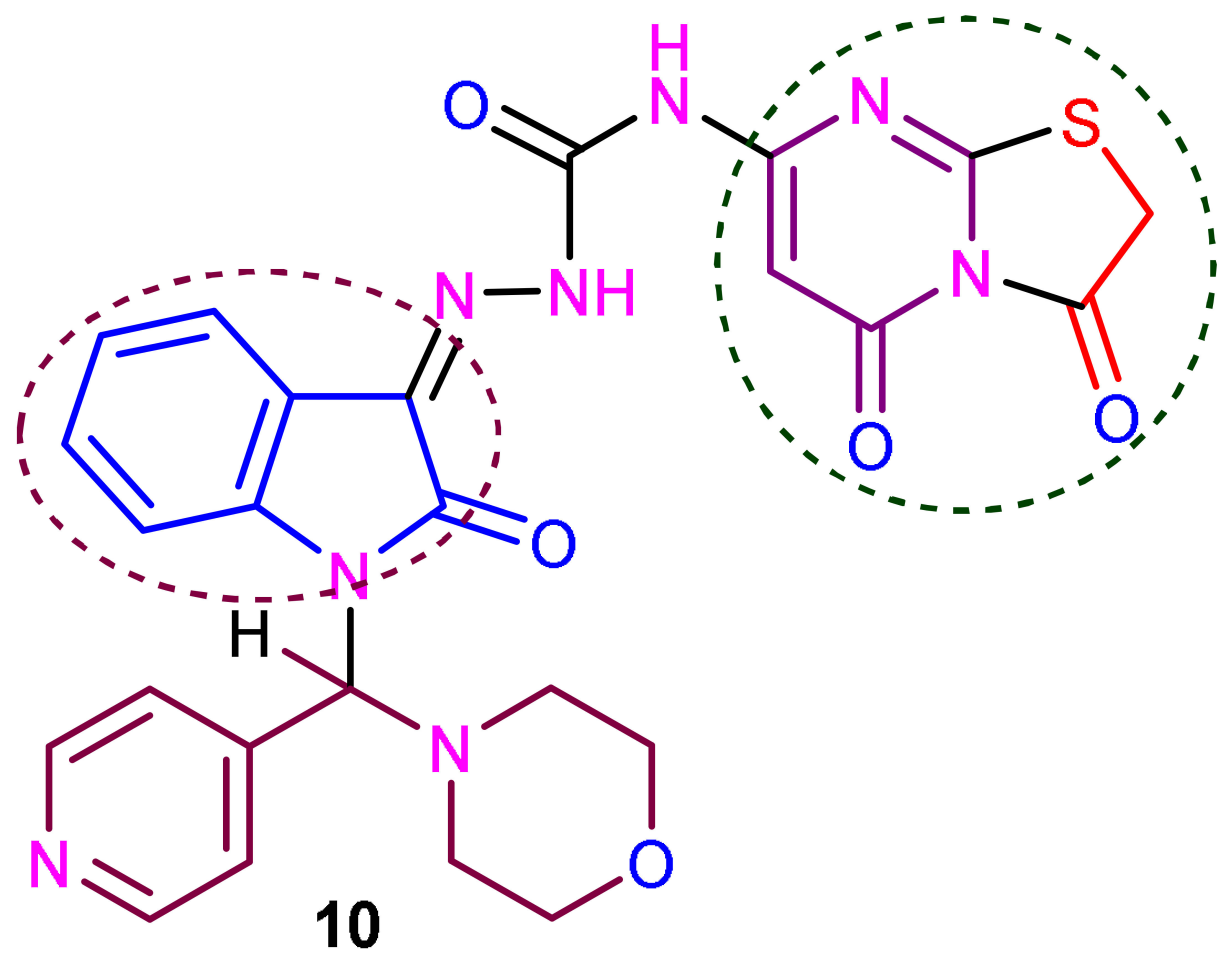	9.8 ± 1.2	9.7 ± 1.1	9.6 ± 1.3	9.5 ± 1.1	0.5
	Isatin, morpholine, pyridine, methyl, hydrazine, carboxamide, thiazolo [3,2-a] pyrimidine, four carbonyl groups, nitrogen, oxygen and sulfur atoms.				
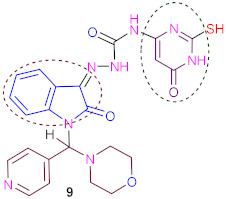	9.9 ± 1.1	9.8 ± 1.2	9.7 ± 1.1	9.6± 1.2	0.6
	Isatin, morpholine, pyridine, methyl, hydrazine, carboxamide, pyrimidine ring, three carbonyl groups, nitrogen, oxygen and sulfur atoms.				
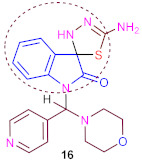	10.1 ± 1.3	10.0 ± 1.4	9.9 ± 1.2	9.8 ± 1.3	0.8
	Isatin, morpholine, pyridine, methyl, amino, spiro [indoline-1,3,4-thiadiazole], carbonyl group, nitrogen, oxygen and sulfur atoms.				
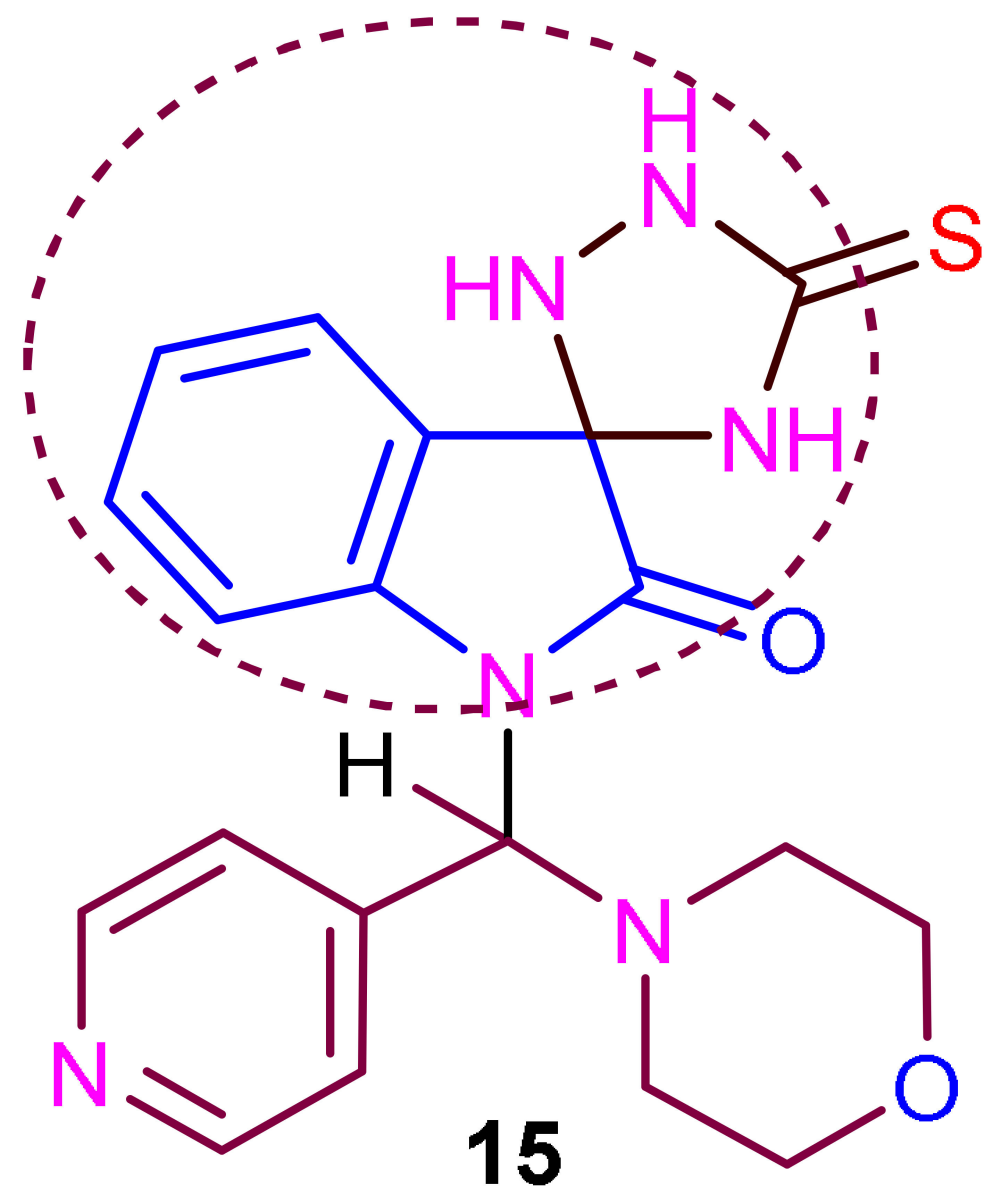	10.3 ± 1.1	10.2 ± 1.2	10.1 ± 1.3	9.9 ± 1.4	0.9
	Isatin, morpholine, pyridine, methyl, thioxo, spiro [indoline-1, 2, 4- triazolidinone], carbonyl group, nitrogen, oxygen and sulfur atoms.				
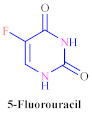	10.7 ± 1.2	10.5 ± 1.1	10.3 ± 1.3	10.1 ± 1.1	0.7
	Pyrimidine ring, two carbonyl groups, nitrogen, oxygen and fluorine atoms.				

^a^**MGC-803** cells are drug-sensitive human gastric carcinoma cells; **MCF-7** cells are drug-sensitive human breast adenocarcinoma cells; **CNE2** cells are drug-sensitive human nasopharyngeal carcinoma cells; and **KB** cells are drug-sensitive human oral carcinoma cells.

## Data Availability

All data are contained within the article and the experimental section.

## Sample Availability

Samples of the synthesized compounds are available from the authors.
